# Grapevine wood microbiome analysis identifies key fungal pathogens and potential interactions with the bacterial community implicated in grapevine trunk disease appearance

**DOI:** 10.1186/s40793-021-00390-1

**Published:** 2021-12-04

**Authors:** Bekris Fotios, Vasileiadis Sotirios, Papadopoulou Elena, Samaras Anastasios, Testempasis Stefanos, Gkizi Danae, Tavlaki Georgia, Tzima Aliki, Paplomatas Epaminondas, Markakis Emmanuel, Karaoglanidis George, Papadopoulou K. Kalliope, Karpouzas G. Dimitrios

**Affiliations:** 1 Laboratory of Plant and Environmental Biotechnology, Viopolis, Department of Biochemistry and Biotechnology, University of Thessaly, 41500 Larissa, Greece; 2 Plant Pathology Laboratory, Faculty of Agriculture, Aristotle University of Thessaloniki, Thessaloniki, Greece; 3 Laboratory of Plant Pathology, Agricultural University of Athens, Iera Odos 75, 11855 Athens, Greece; 4 Laboratory of Mycology, Department of Viticulture, Vegetable Crops, Floriculture and Plant Protection, Institute of Olive Tree, Subtropical Crops and Viticulture, Hellenic Agricultural Organization DIMITRA, 32A Kastorias Street, Mesa Katsabas, 71307 Heraklion, Crete Greece

**Keywords:** Grapevine, Wood microbiome, Trunk diseases, Bacteria, Fungi, Grapevine cultivars, Biogeography

## Abstract

**Background:**

Grapevine trunk diseases (GTDs) is a disease complex caused by wood pathogenic fungi belonging to genera like *Phaeomoniella, Phaeoacremonium, Fomitiporia, Eutypa* and members of the family *Botryosphaeriaceae*. However, the co-occurrence of these fungi in symptomatic and asymptomatic vines at equivalent abundances has questioned their role in GTDs. Hence, we still lack a good understanding of the fungi involved in GTDs, their interactions and the factors controlling their assemblage in vines. We determined the fungal and bacterial microbiome in wood tissues of asymptomatic and symptomatic vines of three main Greek cultivars (Agiorgitiko, Xinomavro, Vidiano), each cultivated in geographically distinct viticultural zones, using amplicon sequencing.

**Results:**

We noted that cultivar/biogeography (lumped factor) was the strongest determinant of the wood fungal microbiome (*p* < 0.001, 22.7%), while GTD symptoms condition had a weaker but still significant effect (*p* < 0.001, 3.5%), being prominent only in the cultivar Xinomavro. Several fungal Amplicon Sequence Variants (ASVs), reported as GTD-associated pathogens like *Kalmusia variispora*, *Fomitiporia* spp., and *Phaemoniella chlamydosporα* (most dominant in our study), were positively correlated with symptomatic vines in a cultivar/viticultural zone dependent manner. Random Forest analysis pointed to *P. chlamydosporα*, *K. variispora*, *A. alternata* and *Cladosporium* sp., as highly accurate predictors of symptomatic vines (0% error rate). The wood bacterial microbiome showed similar patterns, with biogeography/cultivar being the main determinant (*p* < 0.001, 25.5%) of its composition, followed by the GTD status of vines (*p* < 0.001, 5.2%). Differential abundance analysis revealed a universal positive correlation (*p* < 0.001) of *Bacillus* and *Streptomyces* ASVs with asymptomatic vines. Network analysis identified a significant negative co-occurrence network between these bacterial genera and *Phaemoniella, Phaeoacrominum* and *Seimatosporium*. These results point to a plant beneficial interaction between *Bacillus/Streptomyces* and GTD pathogens.

**Conclusions:**

Our study (a) provides evidence that GTD symptomatic plants support a wood fungal microbiome, showing cultivar and biogeography-dependent patterns, that could be used as a proxy to distinguish between healthy and diseased vines, (b) points to strong interactions between the bacterial and fungal wood microbiome in asymptomatic vines that should be further pursued in the quest for discovery of novel biocontrol agents.

**Supplementary Information:**

The online version contains supplementary material available at 10.1186/s40793-021-00390-1.

## Background

Grapevine trunk diseases (GTDs) are considered a major threat for viticulture [1]. However, the ban of sodium arsenate, the main chemical used for their control, along with changes in cultivation practices and the use of infected planting material resulted in a strong upsurge in the incidence of GTDs globally [2–4] with estimated annual economic losses summing to 1.132 million euros [5]. GTDs are considered a complex of diseases that could be distinguished into three major groups: Eutypa, Esca and Botryosphaeria diebacks [6]. Several fungi have been linked with GTDs like: (1) *Eutypa lata* and other *Eutypa* species associated with Eutypa dieback [7], (2) *Phaeomoniella chlamydospora, Phaeoacremonium* spp., and *Fomitiporia mediterranea* associated with esca decline [8, 9] and (3) different genera of *Botryosphaeriaceae* (i.e. *Neofusicoccum, Lasiodiplodia, Diplodia, Botryosphaeria*) associated with the Botryosphaeria dieback [10–12]. GTDs appear mostly in mature vines (> 8 years old) causing central, black necrosis and/or white rot that eventually lead to vine stunting and in acute cases to apoplexy [13]. Foliar symptoms often appear years after infestation [6] and show transitory patterns of appearance [14]. Fungal pathogens have not been recovered yet from symptomatic leaves suggesting that leaf symptoms are associated with the production and translocation to leaves of (1) secondary metabolites produced by the wood pathogens [15–18] (2) phytotoxic by-products of wood decay or phytotoxic metabolites produced by the plant–fungi interaction [19].

Several studies have used culture-dependent approaches to isolate the causal agents of GTDs from symptomatic and comparatively from asymptomatic vines and/or from necrotic or soft rot tissues of vines [3, 5, 20]. These studies suggested that all fungi previously identified as causal agents of GTDs were found at equivalent abundance levels in symptomatic and asymptomatic vines, implying that they are either latent pathogens or members of the endophytic vine microbiome causing symptoms when edaphoclimatic factors and vine genotype are conducive [5]. Culture-dependent approaches and low-resolution molecular tools (i.e. Single-Strand Conformation Polymorphism) failed to unravel the full diversity of the endophytic fungal microbiome in vines [3, 20, 21]. This is due to the limited cultivability of most environmental microorganisms [22] and the bias of culture-dependent methods towards fast growing strains [23]. A few recent studies using amplicon sequencing provided deeper insights into the composition of wood fungal microbiome in GTD symptomatic and asymptomatic vines. For example, Bruez et al. [24] did not identify compositional differences in the communities colonizing non-necrotic tissues of symptomatic and asymptomatic vines. Differences became evident when necrotic tissues were studied comparatively. In those tissues *F. mediterranea* dominated the fungal community. Similar studies in Portugal and Australia did not identify major differences in the fungal microbiome in asymptomatic *vs* symptomatic vines [25, 26]. A recent study by Bruez et al. [27] showed that sodium arsenate application in esca-diseased grapevines resulted in significant changes in the wood fungal microbiome with the most striking result being the strong inhibition of *F. mediterranea*. All these studies focused on a single cultivar in a single vineyard and have not considered differences in the resistance of vine cultivars to GTDs [6, 28] or the role of biogeography in the manifestation of symptoms in vineyards.

Besides fungi, the endophytic bacterial microbiome of grapevine and its role in GTDs is largely unexplored. Grapevines support a diverse bacterial community from roots to berries. The latter have been the focus of most vine microbiome studies [29–31]. Wood-associated bacteria might have a suppressive effect on GTDs. They could act as forerunners of fungal wood pathogens facilitating the infection of wood tissues or as late comers colonizing necrotic or white rot tissues. To date only a few studies have investigated comparatively the composition of the bacterial community in the trunk of asymptomatic and symptomatic vines. Bruez et al. [32] identified *Bacillales, Enterobacteriales* and *Xanthomonadales* as the most abundant bacterial orders colonizing the trunk of grapevines. However, they failed to identify consistent differences in the composition of the bacterial communities in asymptomatic and symptomatic vines. Similar results were obtained in recent studies using amplicon sequencing [24, 26].

We tested the hypotheses that (1) the composition of the fungal wood microbiome differs between asymptomatic and symptomatic vines with GTD-associated fungi being the key drivers of this differentiation, (2) the composition of the bacterial community differs between asymptomatic and symptomatic vines and specific bacterial taxa would be potential interactors with GTD-associated fungi and (3) vine genotype, biogeography and vine GTD condition interactively determine the composition of the vine wood microbiome and the fungal wood pathobiome. These hypotheses were evaluated in asymptomatic and GTD-symptomatic vines from three Greek cultivars, each cultivated in geographically distinct viticultural zones, using amplicon sequencing of the 16S rRNA gene for bacteria and the ITS2 region for fungi. Multivariate analysis and machine learning approaches identified members of the wood fungal microbiome that could act as GTD predictors, and network analysis revealed microbial interactions relevant for the appearance of the disease.

## Methods

### Collection of samples

Wood samples were collected during July–August 2019 from vineyards located in three important and geographically distinct viticultural zones in north-west (Amyntaio), central-south (Nemea, Peloponnese) and southern Greece (Crete) (Additional file 1: Fig. S1). These regions are mainly cultivated with the Greek cultivars Xinomavro, Agiorgitiko and Vidiano, respectively. The three viticultural zones are characterized by different climatic conditions: (1) The Amyntaio region is humid (mean annual precipitation 611 mm) and cool (mean annual temperature 12.2 °C, max 18.4 °C and min 6.7 °C) (2) The Nemea region is equally humid (mean annual precipitation 611.3 mm) but warmer than Amyntaio (mean annual temperature 15.8 °C, max 22.5 °C and min 8.7 °C) (3) The Heraklion region in Crete is the driest (mean annual precipitation 464 mm) and warmest (mean annual temperature 17.8 °C, max 21.9 °C and min 14.0 °C) of the viticultural zones studied.

In each zone, three vineyards, with the age of grapevines varying from 10 to 15 years, were selected for collecting samples. Details on the management practices, age, pruning system and rootstocks used in the nine vineyards of the current study are given in Table 1. In each vineyard, three vines without any visible GTD symptom on leaves and wood, and three vines exhibiting typical foliar GTD symptoms (including leaf discoloration, tiger-stripe chlorosis or necrosis and plant wilting) in the last two years were selected and nominated as asymptomatic and symptomatic vines, respectively. The only exception was recorded for the cultivar Agiorgitiko where for technical reasons asymptomatic plants were sampled only from one of the three vineyards. Each one of the vines selected as symptomatic or asymptomatic were destructively sampled. Specifically, wood samples from the cordons and the trunk of the selected vines were removed using a sterile saw and transferred in the laboratory. Then, cordon and trunk samples were cut longitudinally to identify the extent of necrosis in these wood tissues. Wood samples from asymptomatic plants showing extensive necrotic areas were excluded from further sampling. Overall, samples from symptomatic plants were characterized by extensive necrotic areas, whereas samples from asymptomatic plants were largely free of necrotic areas. Based on this visible inspection, wood chips from necrotic tissues of symptomatic vines and wood chips from non-necrotic tissues of asymptomatic vines were collected using a sterile drill and stored at − 20 °C until further use. White rots were occasionally found adjoining necrotic areas and they were sampled along with necrotic tissues but they were not considered separately. A detailed list of the samples used in the study is given in Additional file 1: Table S1.

**Table 1:** Management practices, pruning practices, age and rootstocs used in the studied vineyards

Vineyard	Cultivar	Management	Training/Pruning	Age (years)	Rootstocs
Amyntaio 1	Xinomavro	Conventional^a^	Double cordon trained, spur pruned	10	1103P
Amyntaio 2	Xinomavro	Conventional	Double cordon trained, spur pruned	10	1103P
Amyntaio 3	Xinomavro	Conventional	Double cordon trained, spur pruned	12	SO_4_
Nemea 1	Agiorgitiko	Conventional	Double cordon trained, spur pruned	15	110 Richter
Nemea 2	Agiorgitiko	Conventional	Double cordon trained, spur pruned	15	110 Richter
Nemea 3	Agiorgitiko	Conventional	Double cordon trained, spur pruned	12	110 Richter
Herakleion 1	Vidiano	Conventional	Double cordon trained, spur pruned	10	110 Richter
Herakleion 2	Vidiano	Conventional	Double cordon trained, spur pruned	15	110 Richter
Herakleion 3	Vidiano	Conventional	Double cordon trained, spur pruned	13	110 Richter

^a^Conventional type of management involves application of fungicides and insecticides with the active ingredients used varying largely between viticultural zones and even vineyards

### DNA extraction

DNA from the wood samples was extracted using the Cetyltetramethyl ammonium bromide (CTAB) method [33] with slight modifications. Briefly, 250 mg of the collected wood tissue were homogenized in a chilled pestle and mortar using liquid nitrogen. 1200 μl (600 μl in two doses) of 2X CTAB extraction buffer (100mMTris-HCl, 20 mM EDTA, 1.4 M NaCl, 2% CTAB, 0,5% v/v β-mecraproethanol) were added and homogenized. The samples were incubated at 65 °C for 30–60 min with occasional vortexing. Samples were centrifuged at 10,000 rpm for 10 min and the supernatant (~ 400 μl) was transferred to new tubes. 600 μl of chloroform:isoamyl alcohol (24:1) were added and mixed by inverting the tube several times. Samples were centrifuged at 13,000 rpm for 15 min. The aqueous phase was transferred into a new tube and 600 μl of chloroform were added. The tubes were centrifuged at 13,000 rpm for 10 min. Then, 1 volume of chilled isopropanol was added, followed by quick and gentle inversion and incubated at − 20 °C for 1 h. The DNA pellet was precipitated at 13,000 rpm for 20 min, washed twice with 70% ethanol and precipitated at 13,000 rpm for 3 min. The DNA pellet was then suspended in 50 μl of pre-heated TE-buffer. DNA integrity was initially assessed via agarose gel (0.8%) electrophoresis and DNA concentrations were determined using the Quant-iT kit with a Qubit Fluorometric device (Invitrogen, USA).

### Amplicon sequencing analysis

The bacterial and fungal wood microbiome of vines was determined via amplicon sequencing of the V4 region of the 16S rRNA gene and the ITS2 region respectively, using a HiSeq, Illumina platform in Rapid Mode 2 × 250 paired-end in Admera Health company (New Jersey, USA). Amplification, facilitating sample-wise multiplexing during sequencing, was based on our in-house protocol described in detail in Vasileiadis et al. [34]. Briefly, extracted DNA was diluted to a final concentration of 1 ng μl^−1^ for fungi and 0.2 ng μl^−1^ for bacteria. We followed a two-step amplification process, which allowed indexing of our samples. In the first step, the bacterial 16S rRNA gene was amplified with the updated version of the primers 515f-806r [35] following the protocol of the earth microbiome project [36]. Regarding fungi, a first amplification step of the ITS2 region was performed with primers ITS7F-ITS4R [37, 38]. The first amplification step included 28 cycles using the primers mentioned above, followed by a second PCR of 7 cycles using the same primers with sample-specific 5’ overhangs. All PCR reactions were conducted with the use of Q5® High-Fidelity DNA Polymerase (NEB, Ipswich, Massachusetts, USA). Primers, PCR conditions and the reagents used are given in Additional file 1: Tables S2 and S3 respectively. Amplicon libraries were cleaned up with the use of NucleoMag® NGS Clean-up and Size Select Kit (Macherey‐Nagel, Duren, Germany) according to manufacturer instructions and they were sent for sequencing. Amplification of non-template controls gave no amplicons and their sequencing provided particularly low number of reads, hence they were not considered in the analysis.

### Bioinformatic analysis

Sequence demultiplexing to their samples of origin was performed with flexbar v3.0.3 [39] not allowing any barcode mismatches. Sequences were then quality-trimmed/filtered and checked for chimerical sequences with the dada2 v1.14.1 [40] package of the R software v.4.0.2 [41]. Amplicon sequence variants (ASVs) were assembled and taxonomically assigned using the Silva v.138 [42] and UNITE ITS v.8.2 (04.02.2020) [43] reference databases for bacteria and fungi, respectively. ASVs not classified at Kingdom and Phylum level were excluded from downstream analysis. Whereas we included in the analysis ASVs with 80% or higher taxonomy annotation bootstrap confidence in the domain/kingdom taxa as previously suggested [44]. ASV classifications are provided at the lowest taxonomical rank passing this threshold.

### Statistical analysis

The ASV matrices of bacteria and fungi were used to assess the effects of vine GTD condition and the lumped factor biogeography/cultivar on the β-diversity of the wood microbiome. Non-metric multidimensional scaling (NMDS) and permutational multivariate analysis of variance (PERMANOVA) were used to assess β-diversity differences for bacterial and fungal communities between symptomatic and asymptomatic vines either collectively for all cultivars/viticultural zones or separately for each viticultural zone/cultivar. NMDS was performed with dissimilarity matrices using the Bray–Curtis algorithm, while the association of microbiome composition with the covariates of interest and the determination of the % of variance explained by those factors were accessed with PERMANOVA [45] using 999 permutations as implemented in the Vegan v2.5–7 [46] package of the R software. Differential abundance (DA) tests, using the Kruskal–Wallis and the Wilcoxon rank-sum post-hoc tests, were employed to identify bacterial and fungal taxa that were significantly associated with symptomatic and asymptomatic vines. P-value adjustment was conducted with false discovery rate (FDR) algorithm with implemented adjusted P-value cut-off values of 0.05. Heatmaps were produced to depict ASVs whose relative abundance was positively correlated with symptomatic or asymptomatic vines according to the DA tests.

The GTD-associated fungal genera and the fungal and bacterial genera that participate in 10% of the samples with at least 1% relative abundance were used in network analysis aiming to identify positive or negative co-occurrence networks. Spearman correlation tests were performed among microbial genera and the data obtained were used as adjacency matrix for the weighted network analysis performed with the igraph v1.2.6 [47] package of the R software. The minimum spanning tree algorithm was used for filtering the adjacency matrix choosing the shortest possible combination of connections among tree-nodes on the generated undirected networks [48]. Afterwards, the sub-community clusters were identified according to local densities. The force-directed Fruchterman-Reingold layout [49] was used for plotting the final network.

We further selected fungal ASVs to define potential predictors of the GTD status of grapevines via Random Forest analysis. The data matrix was initially subsampled with replacement to equal read numbers (rarefied) as suggested by Weiss et al. [50]. Then, a series of Random Forest supervised classification models were performed by the pime v0.1.0 R-software package [51], which relies on the randomForest v4.6.14 R-software package [52]. Briefly, low prevalence taxa were filtered out for a range of minimum prevalence values (from 5 to 65% at 5% increments) for each sample. Each Random Forest model was run with 1000 trees, using 66% of the samples for building the model and 33% of the samples as out of bag (OOB) samples for the model error assessment. The OBB rate for each prevalence level was calculated for the dataset and the best compromise between model error rate and the remaining dataset reads was selected. The Random Forest analysis was implemented on a dataset of 63 samples (18 from asymptomatic and 45 from symptomatic vines) and 1182 taxa. Overall, the 40% of minimum prevalence cutoff was selected as the best compromise between model error and ASV/sequence numbers for the model, corresponding to 118,161 sequences and 22 ASVs, with an internal OOB error rate 6.25%. Following the Random Forest-based pime approach, the Kruskal–Wallis non-parametric ANOVA equivalent test coupled with a post hoc Wilcoxon rank sum analysis was performed for further filtering out ASVs showing no significant differences between GTD sample groups. This reduced the dataset size to 10 ASVs and 50,649 sequences, which was re-tested for its sample classification accuracy with Random Forests according to the aforementioned parameters.

All data figures for the analysis were produced with the R package ggplot2 v3.3.3 [53]. The sequencing data were submitted to Sequence Read Archive of NCBI with bioproject accession number PRJNA738455. The full analysis code can be found in the Github repository in the link: https://github.com/Fotisbs/Grapevine_wood_microbiome-.git

## Results

### Fungal microbiome

#### Composition of the wood fungal microbiome

In total 1,043,027 non chimeric quality fungal sequences (sequence numbers in the analyzed samples ranged from 1413 to 33,223) were obtained and they were assigned to 1378 ASVs. Rarefaction curves reached a plateau in all samples suggesting that our sequencing effort adequately covered the diversity of endophytic fungi in vines (Additional file 1: Fig. S2a). Overall, the fungal wood microbiome was composed of *Ascomycota* (75.2–97.6%, mean 88.7%) and *Basidiomycota* (2.4–23.3%, mean 10.8%). In total 31 fungal Classes were identified with the most abundant being *Dothideomycetes* (33.9–77.2%, mean 46.2%), *Sordariomycetes* (15.9% – 32.8%, mean 21.8%), *Eurotiomycetes* (1.3–21.2%, mean 13%), *Agaricomycetes* (0.2–20.9%, mean 8.1%), *Leotiomycetes* (0.1–18%, mean 4.6%) and *Lecanoromycetes* (0.3–4.1%, mean 2%) summing to a total relative abundance of 93.5–97.6% (Additional file 1: Fig. S3).

#### Factors structuring the wood fungal microbiome of vines

**Fig. 1:**
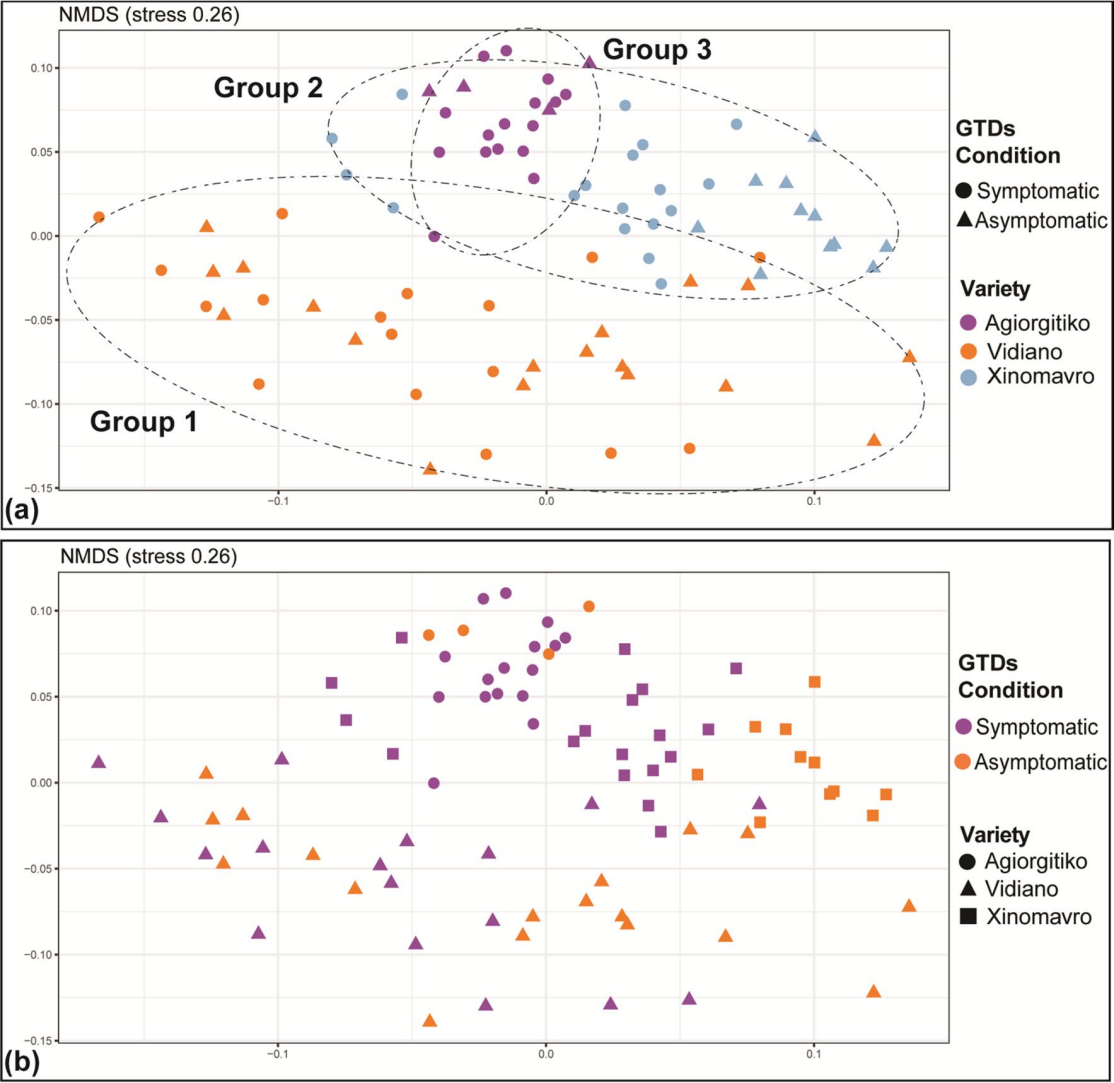
NMDS ordination of wood samples, analyzed for their fungal microbiome, according to their location/cultivar (Agiorgitiko, Xinomavro, Vidiano) (**a**) and their GTD status (asymptomatic vs symptomatic) (**b**)

Preliminary analysis of the amplicon sequencing data suggested a non-significant effect of the type of plant tissue studied (cordon or trunk) on the fungal community and hence, it was not considered as an explanatory variable in our analysis. When samples from all viticultural zones/cultivars were considered, NMDS analysis showed a clear grouping (*p* < 0.01) of the samples according to viticultural zone/cultivar (Fig. 1a), but no clustering according to their GTD condition (symptomatic vs asymptomatic plants) (Fig. 1b). Still, PERMANOVA showed that GTD condition and viticultural zone/cultivar had a statistically significant effect and explained 3.5% (*p* < 0.001) and 22.7% (*p* < 0.001) of the dataset variance, respectively (Additional file 1: Table S4).

**Fig. 2:**
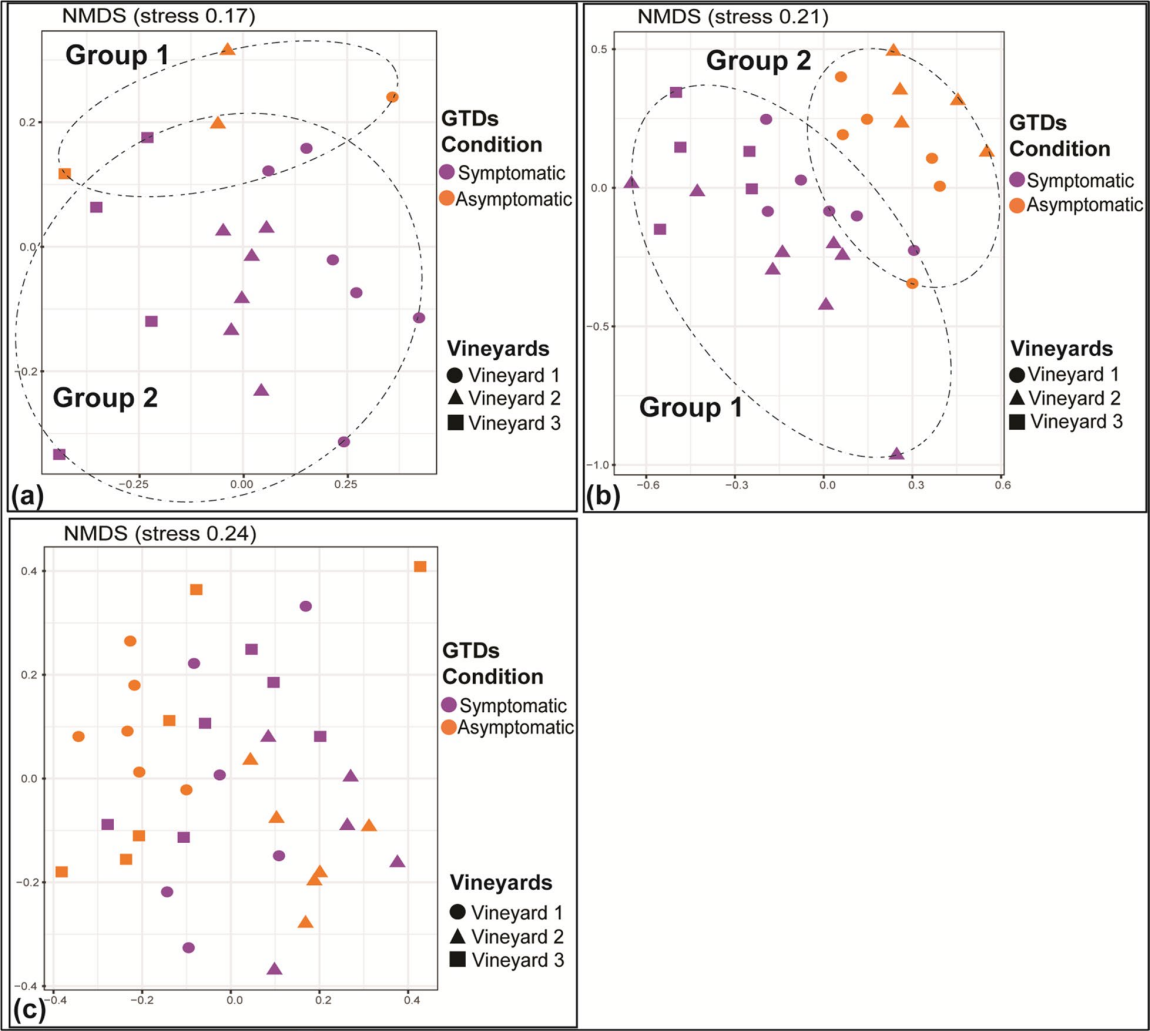
NMDS ordination of the fungal microbiome in wood samples collected from different locations/cultivars like: **a** Agiorgitiko **b** Xinomavro **c** Vidiano and analyzed according to their GTD status (asymptomatic vs symptomatic)

We further analyzed the differences in the β-diversity of the fungal microbiome imposed by GTDs separately for each genotype/viticultural zone. Regarding Xinomavro, NMDS analysis showed a clear separation of samples collected from GTD-asymptomatic and -symptomatic plants (Fig. 2b). Further PERMANOVA showed that GTD condition was the most significant factor (*p* < 0.001) explaining 13.9% of the variance, while vineyard topography (in the same viticultural zone) contributed 8.6% (*p* < 0.05) (Additional file 1: Table S4). In contrast, NMDS analysis for the cultivars Agiorgitiko and Vidiano showed a marginal (*p* < 0.05) or not clear separation (*p* > 0.05) respectively, of the symptomatic and asymptomatic samples (Fig. 2a, c). PERMANOVA further indicated that the GTD condition of plants explained 8.4% and 4.2% of the variance for Agiorgitiko and Xinomavro respectively and had a marginally significant (*p* < 0.05) or non-significant (*p* > 0.05) effect on the fungal β-diversity. Still for these two cultivars, the vineyard location (within the same viticultural zone) was the most significant factor (*p* < 0.001) explaining 20.5% and 15% of the variance in each case (Additional file 1: Table S4).

#### Fungi of the wood microbiome associated with symptomatic and asymptomatic vines

**Fig. 3:**
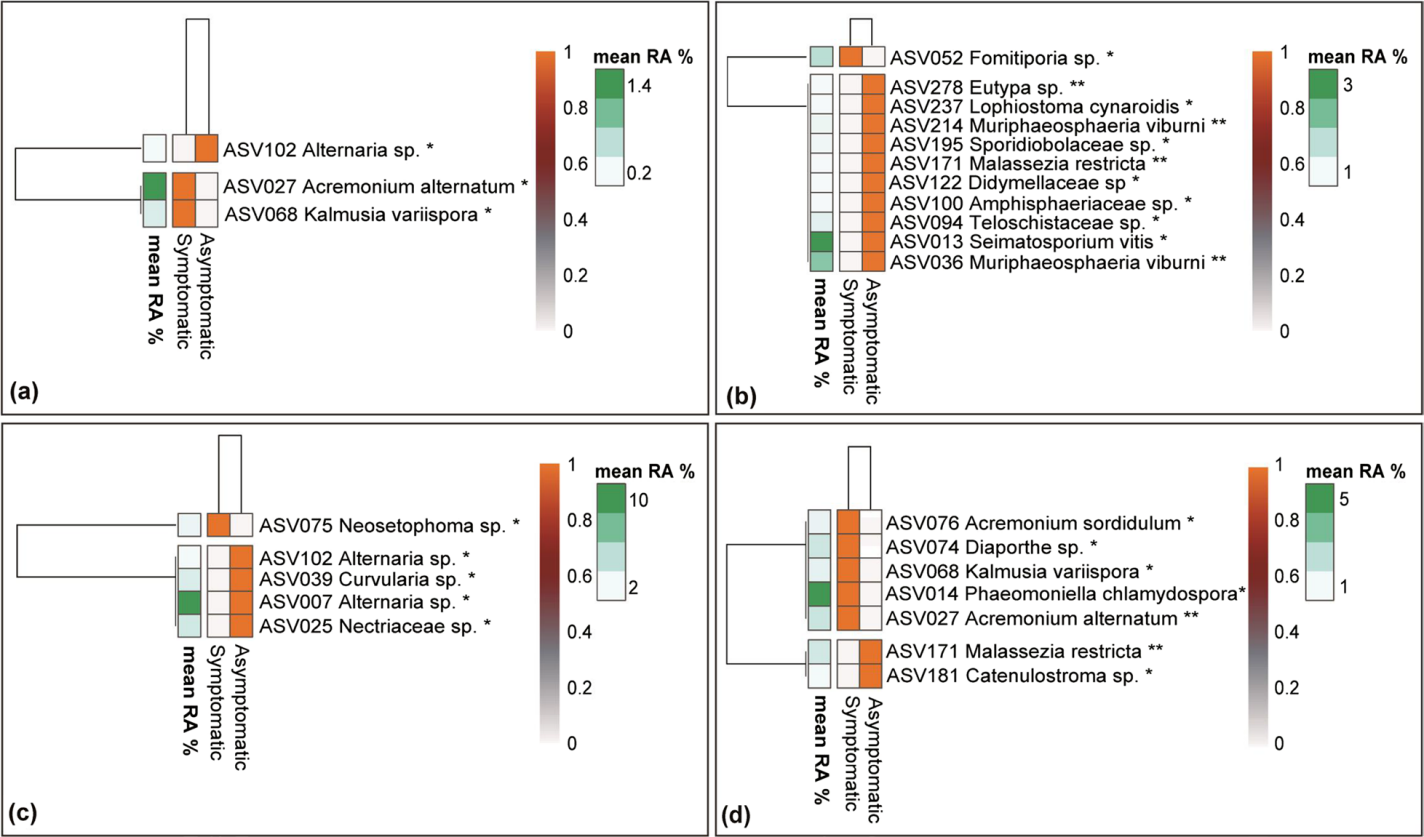
Differential abundance (DA) heatmaps presenting fungal ASVs whose relative abundance showed a significant positive correlation (*p* < 0.05*, *p* < 0.01**) with the wood samples from vines with (symptomatic) or without (asymptomatic) GTD symptoms. Results are presented collectively for all cultivars/locations (**a**) and separately for each variety/location (**b**) Agiorgitiko (**c**) Xinomavro and (**d**) Vidiano

We further attempted to define ASVs whose relative abundance significantly differs between asymptomatic and symptomatic plants. DA heatmaps were constructed collectively for all cultivars/viticultural zones and separately for each cultivar/viticultural zone. When all cultivars were considered together only three ASVs showed significant association with either asymptomatic or symptomatic vines (Fig. 3a). ASV102 assigned to the genus *Alternaria,* showed a consistently higher relative abundance in asymptomatic compared to symptomatic plants. Conversely, ASV027 and ASV068, assigned to *Acremonium alternatum* and *Kalmusia variispora* respectively, showed significantly higher relative abundance in the symptomatic vines.

We expanded our analysis and looked separately in each cultivar/viticultural zone for fungal ASVs with differential abundance patterns in symptomatic *vs* asymptomatic plants. Regarding the cultivar Agiorgitiko, we identified 11 ASV (*p* < 0.05) that showed differential abundance between asymptomatic and symptomatic vines. Amongst them, ASVs belonging to *Eutypa* spp., *Lophiostoma cynaroidis*, *Sporidiobolaceae, Malassezia restricta*, *Didymellaceae, Amphisphaeriaceae, Teloschistaceae*, *Seimatosporium vitis* and *Muriphaeosphaeria viburni* were associated with asymptomatic plants, unlike AS0V52, assigned to the genus *Fomitiporia,* which was amongst the most abundant ASVs of the wood microbiome, and it was systematically associated with symptomatic plants (Fig. 3b). Regarding the cultivar Xinomavro, we identified a single ASV, assigned to genus *Neosetophoma,* that was associated with symptomatic vines (Fig. 3c)*.* In contrast, ASVs belonging to the genera *Alternaria*, *Curvularia* and the family *Nectriaceae* were associated with asymptomatic vines. In the cultivar Vidiano, we identified ASVs belonging to *P. chlamydospora*, one of the most dominant ASVs in our dataset (ASV014), *Acremonium sordidulum*, *Diaporthe* spp., *K. variispora* and *A. alternatum,* all associated (*p* < 0.05) with symptomatic vines (Fig. 3d), compared to ASVs belonging to *M. restricta* and *Catenulostroma* spp., which showed a consistent association with asymptomatic vines.

#### Differential abundance of fungal ASVs belonging to genera associated with GTDs

We further focused our analysis on fungal ASVs with relative abundance > 0.01% that are taxonomically assigned to genera/species previously identified as causal agents of GTDs like *P. chlamydospora, Phaeoacremonium* spp., *Fomitiporia* spp., *Diplodia* spp., *Phellinus rhamni, Seimatosporium vitis, K. variispora, Dothiorella rosulata, Neofusicoccum* spp., *Eutypa* spp., *Botryosphaeria* spp., *Lasiodiplodia* spp., *Neofabrea* spp., *Didymosphaeria* spp., *Diaporthe* spp. In total 61 ASVs were identified and used in the analysis with their relative abundance ranging from 0.03 to 12.9% (Additional file 1: Table S5). The differential abundance of these fungal ASVs in symptomatic and asymptomatic vines was statistically compared collectively for all geographical locations/cultivars together and separately (Additional file 1: Fig. S4). When data for all cultivars/viticultural zones were considered together, *P. chlamydospora*, (ASV014, *p* < 0.01) and *K. variispora* (ASV068, *p* < 0.001) showed significantly higher relative abundance in symptomatic *vs* asymptomatic vines (Additional file 1: Fig. S4a). When data for each cultivar/viticultural zone were analyzed separately, in the cultivar Agiorgitiko we identified two ASVs belonging to *S. vitis* (ASV013 and ASV338, *p* < 0.01) that showed significantly higher relative abundance in asymptomatic vines, in contrast to a *Fomitiporia* ASV (*p* < 0.01), which prevailed in the symptomatic vines (Additional file 1: Fig. S4b). For the cultivar Xinomavro (Additional file 1: Fig. S4c), we identified six ASVs all showing increased relative abundance in symptomatic vines including *P. chlamydospora* (*p* < 0.001), *Phaeoacremonium iranianum* (*p* < 0.001), *K. variispora* (*p* < 0.01), *Neosetophoma* spp., (ASV075 and ASV159, *p* < 0.01). Finally, for Vidiano, five ASVs showed significantly higher relative abundance in symptomatic *vs* asymptomatic vines. They were assigned to *P. chlamydospora* (*p* < 0.01), *K. variispora* (*p* < 0.01) and *Diaporthe* spp., (*p* < 0.01) (Additional file 1: Fig. S4d).

### Wood bacterial microbiome

#### Composition of the wood bacterial microbiome of vines

In total 1,632,972 quality sequences for bacteria (sequence numbers in the analyzed samples ranged from 3318 to 51,768) were obtained and they were assigned to 7908 ASVs. Rarefaction curves reached a plateau in all samples suggesting that our sequencing effort adequately covered the diversity of endophytic bacteria in vines (Additional file 1: Fig. S2b). Overall, the wood bacterial microbiome was composed of 30 phyla. *Proteobacteria* (24.8–77.4%, mean relative abundance 49.5%), *Actinobacteriota* (11.4–22.8%, mean 17.6%), *Bacteroidota* (5.5–20.8%, mean 13.4%), *Firmicutes* (0.4–49.4%, mean 9.3%), *Planctomycetota* (0.1–5.4%, mean 1.9%), *Abditibacteriota* (0.7–2.4%, mean 1.4%), *Verrucomicrobiota* (0.2–3.2%, mean 1.4%), *Acidobacteriota* (0.2–2.8%, mean 1.1%) and *Chloroflexi* (0.02–1.84%, mean 0.8%) were the most dominant phyla, all summing to a relative abundance of 94.4–99.3% (Additional file 1: Fig. S5a). Regarding *Proteobacteria, α-* (14.4–69.8%, mean relative abundance 39.9%) and *γ-Proteobacteria* (5.3–15%, mean 9.5%) were the most dominant classes. An interesting observation was the dominance of Firmicutes in the asymptomatic vines of the cultivar Xinomavro.

#### Factors structuring the wood bacterial microbiome

**Fig. 4:**
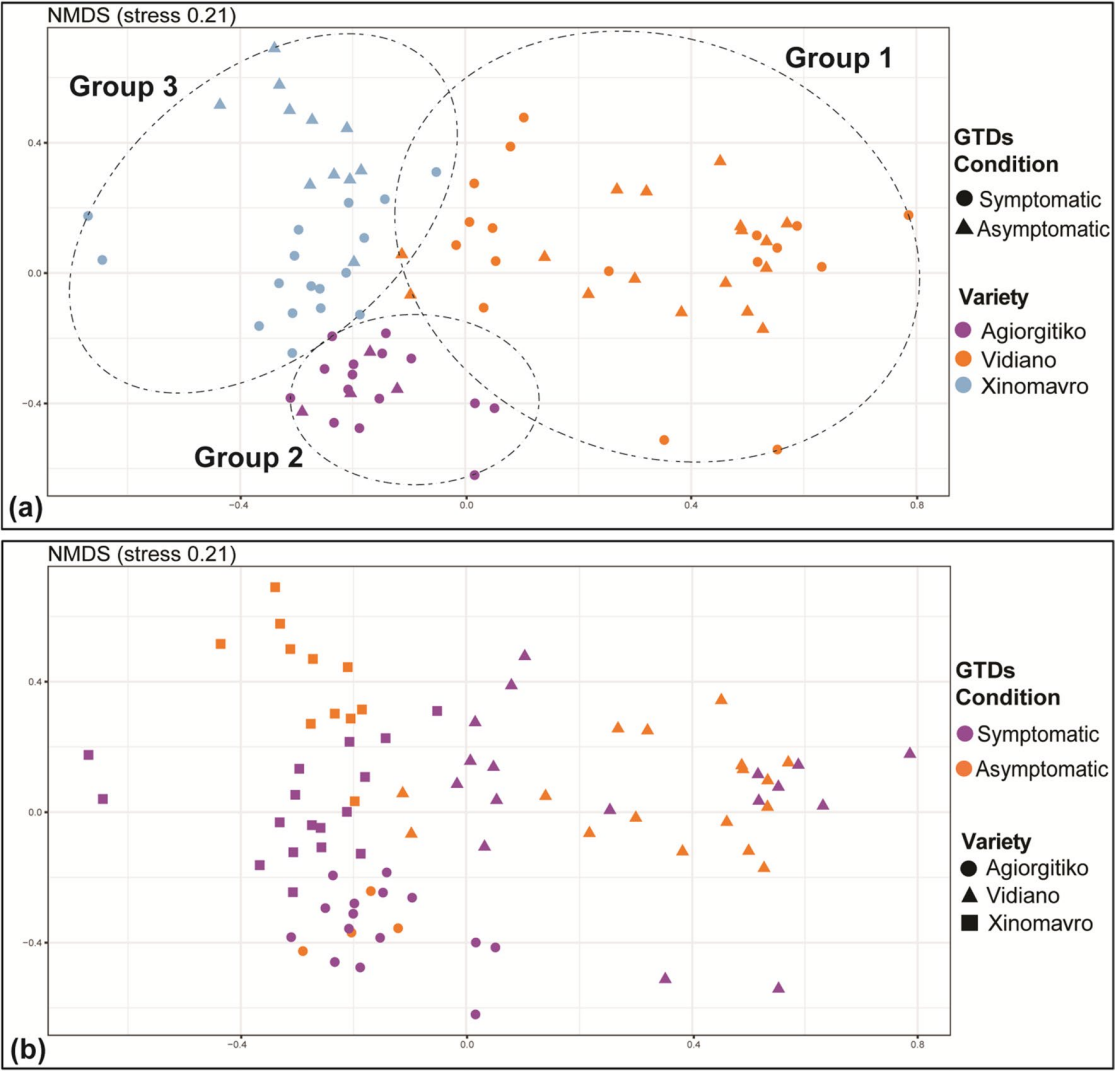
NMDS ordination of wood samples, analyzed for their bacterial microbiome, according to their location/cultivar (Agiorgitiko, Xinomavro, Vidiano) (**a**) and their GTD status (asymptomatic vs symptomatic) (**b**)

In line with the fungal wood microbiome, we observed that the two plant parts sampled, cordons and trunk, supported similar bacterial communities and hence this factor was not considered further in our analysis. When samples from all viticultural zones/cultivars were considered, NMDS analysis showed a clear grouping (*p* < 0.01) of the samples according to viticultural zone/cultivar (Fig. 4a), whereas no clustering according to the GTD conditions of samples (symptomatic vs asymptomatic) was observed (Fig. 4b). Still PERMANOVA showed that GTD condition and cultivar/viticultural zone exerted a statistically significant effect (*p* < 0.001) on bacterial community structure explaining 5.2 and 25.5% of the dataset variance respectively (Additional file 1: Table S4).

**Fig. 5:**
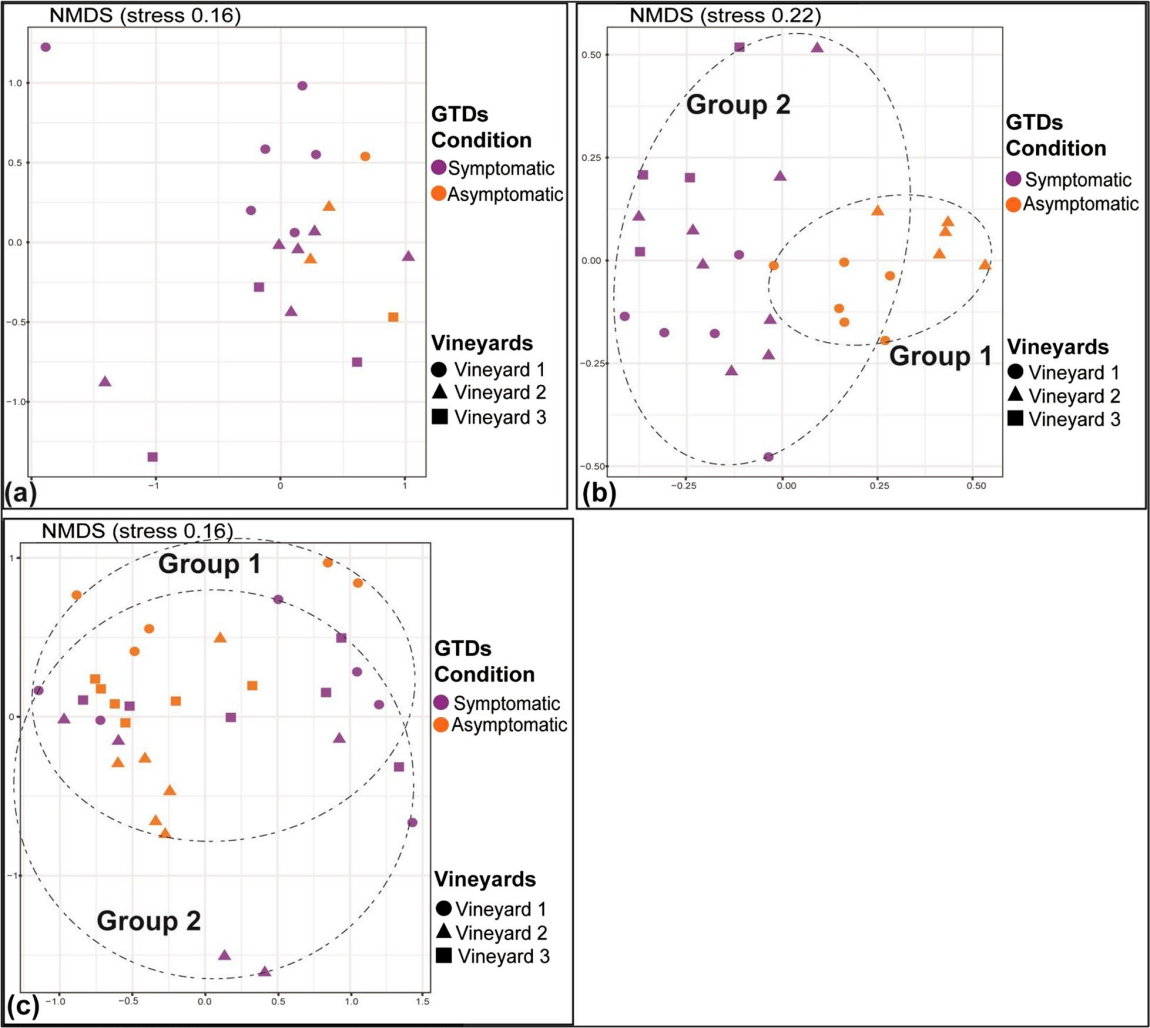
NMDS ordination of the bacterial microbiome in wood samples collected from different locations/cultivars like: (**a**) Agiorgitiko (**b**) Xinomavro (**c**) Vidiano and analyzed according to their GTD status (asymptomatic vs symptomatic)

We further analyzed our data separately for each viticultural zone/cultivar aiming to explore further the effect of GTD occurrence on the composition of the bacterial microbiome. Regarding the cultivar Xinomavro, NMDS analysis showed a clear separation of the samples from symptomatic and asymptomatic vines (*p* < 0.01) (Fig. 5b). PERMANOVA verified the significant effect of the GTD plant condition on the composition of the wood bacterial community (*p* < 0.001), which explained 19.7% of the variance, whereas vineyard topography (within the viticultural zone of Amyntaio) did not have a significant effect (*p* > 0.05) (Additional file 1: Table S4). Regarding the other two cultivars, we noted a weak clustering of the samples according to their GTD status only in the case of Vidiano (*p* < 0.05) (Fig. 5c). PERMANOVA analysis revealed that GTD condition and vineyard topography (at the viticulture zone scale) explained 5.3% (*p* > 0.05) and 13.6% (*p* < 0.05) of the variance respectively for Agiorgitiko, and 6% (*p* < 0.01) and 12.1% (*p* < 0.01) for Vidiano (Additional file 1: Table S4).

#### Wood microbiome bacteria associated with symptomatic and asymptomatic vines

**Fig. 6:**
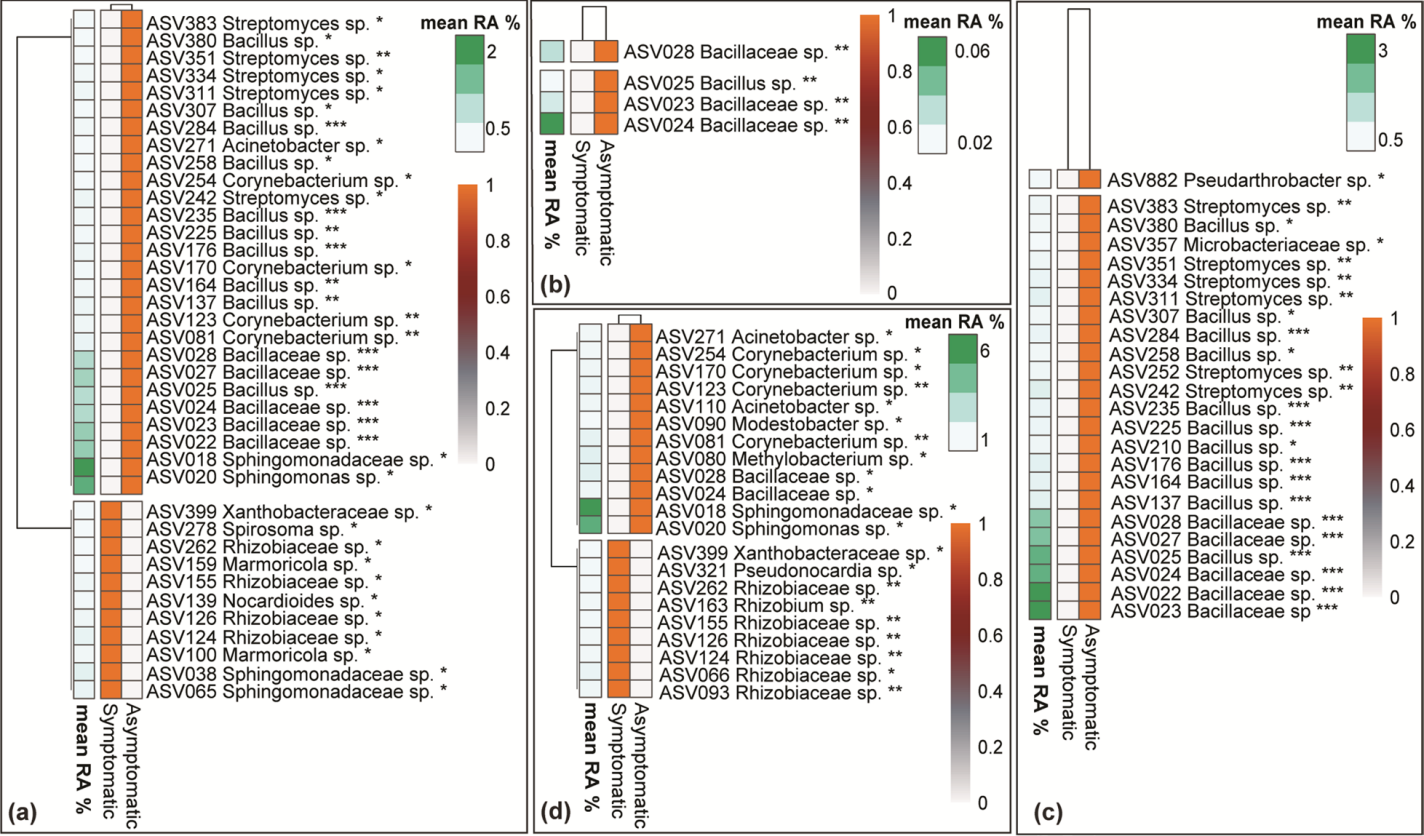
Differential abundance (DA) heatmaps showing bacterial ASVs whose relative abundance showed a significant positive correlation (*p* < 0.05*, *p* < 0.01**, *p* < 0.001***) with the wood samples from vines with (symptomatic) or without (asymptomatic) GTD symptoms. Results are presented collectively for all cultivar/locations (**a**) and separately for each cultivar/location (**b**) Agiorgitiko (**c**) Xinomavro and (**d**) Vidiano

DA heatmaps pointed to ASVs that were consistently associated with symptomatic or asymptomatic vines. When data from all viticultural zone/cultivars were considered, we identified several ASVs, mostly belonging to *Rhizobiaceae,* that showed consistently higher relative abundance in symptomatic vines (Fig. 6a). On the other hand, five *Streptomyces*, ten *Bacillus,* five *Bacillaceae,* and four *Corynebacterium* ASVs were consistently present in the asymptomatic vines. When the same analysis was conducted separately for each viticultural zone/cultivar, we identified four ASVs, all belonging to *Bacillaceae*, that were associated (*p* < 0.05) with asymptomatic vines of the cultivar Agiorgitiko (Fig. 6b). Similarly**,** we noted five *Bacillaceae*, eleven *Bacillus* and six *Streptomyces* ASVs that were systematically associated (*p* < 0.05) with asymptomatic vines of the cultivar Xinomavro (Fig. 6c). Regarding, Vidiano six ASVs belonging to *Rhizobiaceae* were systematically associated with symptomatic vines (Fig. 6d), whereas ASVs belonging to *Sphingomonadaceae* (2), *Corynebacterium* (4), *Bacillaceae* (2) and *Acinetobacter* (2) were associated with asymptomatic vines.

### Network analysis of the wood microbiome

Network analysis within the fungal microbiome across all cultivars revealed a significant negative co-occurrence pattern between *Alternaria* and the two GTD-linked fungal genera *Phaeomoniella* and *Phaeoacremonium* in the asymptomatic but not in the symptomatic vines (Additional file 1: Fig. S6). Network analysis between the GTD-associated fungi and the bacterial microbiome in all cultivars revealed a significant negative co-occurrence pattern between the GTD-relevant fungal genera *Phaeomoniella* and *Seimatosporium* with *Bacillus,* and of *Phaeoacremonium* with *Streptomyces*, only in the asymptomatic grapevines (Additional file 1: Fig. S6).

### Random forest analysis of the fungal wood microbiome

**Fig. 7:**
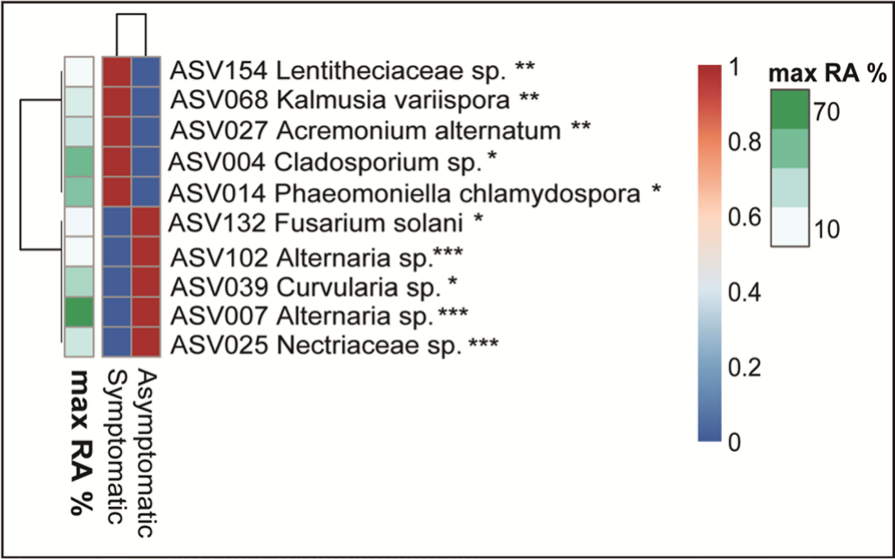
Random Forest heatmap showing rarefied ASVs whose max relative abundance showed a significant positive correlation (*p* < 0.05*, *p* < 0.01**, *p* < 0.001***) with the wood samples from vines with (symptomatic) and without (asymptomatic) GTD symptoms

To further explore our fungal dataset for possible marker ASVs for asymptomatic and symptomatic samples, a Random Forest supervised learning approach was used. This analysis showed that the fungal microbiome had strong discriminative power and pointed to 10 ASVs that could be considered as predictors of the GTD status of the vines. Hence ASVs belonging to *Cladosporium* spp., (ASV004, *p* < 0.05, most dominant), *P. chlamydosporα* (ASV014, *p* < 0.05, dominant), *K. variispora* (ASV068, *p* < 0.001), *A. alternatum* (ASV027, *p* < 0.01) and *Lentitheciaceae* (ASV154, *p* < 0.01) were all predictors of symptomatic plants (Fig. 7). Whereas ASVs belonging to *Fusarium solani* (ASV132, *p* < 0.038), *Alternaria* spp., (ASV102 *p* < 0.0 and ASV007 *p* < 0.001), *Curvularia* spp., (ASV039, *p* < 0.02) and *Nectriaceae* (ASV025, *p* < 0.001) were highlighted from the model as identifiers of asymptomatic vines. These ASVs had an overall (for both symptomatic and asymptomatic samples) OOB error rate of 7.94% (Additional file 1: Fig. S7). When using the five ASV identifiers for symptomatic samples we noted a high classification accuracy with OOB error rates of 0% suggesting that all 45 symptomatic samples were classified correctly. In contrast, we observed a low classification accuracy when using the ASV indicators of asymptomatic plants with an OOB error rate of 27%.

## Discussion

We determined the composition of the wood fungal microbiome in symptomatic and asymptomatic vines from three different Greek cultivars, each located in a geographically distinct viticultural zone, aiming to identify consistent drivers of the disease. In this quest we used amplicon sequencing approaches and relevant statistical and machine learning tools to identify potential biotic drivers of GTDs. We further expanded our study to the bacterial wood microbiome expecting to identify interactors with the fungal microbiome.

### Fungal microbiome

The wood fungal microbiome of all studied cultivars was dominated by *Ascomycota*, while *Basidiomycota* constituted a substantially smaller fraction with *Agaricomycetes* being the most prevalent amongst them. Similar compositional patterns, denoting a clear dominance of *Ascomycota* in the wood microbiome of vines, have been reported in most previous studies [24, 25, 31]. An interesting pattern was the consistent and significant (*p* < 0.05) increase in the contribution of *Agaricomycetes* in symptomatic vines in all studied cultivars. This observation might be associated with the capacity of members of this fungal taxon to induce white rots in wood tissues, including *Fomitiporia* spp., a key colonizer of white rot tissues in vine exhibiting GTD symptoms [1]. Recently, Paolinelli et al. [54] suggested that the increase in the *Basidiomycota/Ascomycota* ratio observed in symptomatic over asymptomatic vines could be used as an early warning for the establishment of GTDs in vines.

We first explored the factors shaping the wood fungal microbiome and identified viticultural zone and/or cultivar, considered as a lump factor based on our experimental setup, as the main determinant of the fungal community. In contrast, we noted a weak differentiation of the wood fungal microbiome between symptomatic and asymptomatic samples. To date several studies have explored the effect of biogeography, cultivar and vintage on the grapevine microbiome focusing on above-ground (berries, leaves) [29, 55, 56] and/or below-ground plant associated compartments (soil, rhizosphere, roots) [30, 57], while the trunk microbiome has attracted little attention. These studies have identified biogeography as a main determinant of the non-random assemblage of the fungal vine microbiome, especially when microbiomes are looked at the macro-geographical scale, whereas at regional scale (micro-geographical scale) other confounding factors like cultivar and vintage become important [29]. The wood fungal microbiome, as determined in our study, seems to share assemblage mechanisms with the other grapevine compartments. Although we could not distinguish between cultivar and biogeography, we speculate that the latter has possibly a stronger effect in shaping the fungal wood microbiome considering the wide geographical scale of our study. The limited dissimilarity of the fungal microbiome between asymptomatic and symptomatic vines, when the viticultural zone/cultivar factor was not considered, is in line with the previous studies, which, following similar sampling strategies, did not identify significant differences between symptomatic and asymptomatic vines [3, 20, 25, 26].

We further determined the differences in the fungal wood microbiome separately for each cultivar/viticultural zone, taking under consideration the variable climatic conditions that prevail in the studied viticultural zones and the different sensitivity of the studied cultivars to GTDs. Indeed, Agiorgitiko and Vidiano are more susceptible to GTDs compared to the more resistant Xinomavro [28]. We noted that the three cultivars exhibited different responses in their assembly of the fungal microbiome in symptomatic and asymptomatic vines. Hence, Xinomavro was the only cultivar that assembled clearly distinct endophytic fungal communities in symptomatic *vs* asymptomatic plants. This result may be due to the clear visual distinction between potentially healthy and diseased plants, which is facilitated by the higher resistance of the cultivar Xinomavro to GTDs. Still, we could not exclude the contribution of the local climatic conditions, that varied in the three viticultural zones studied (see Methods), on the differential response of the studied cultivars to the assemblage of the wood microbiome in asymptomatic and symptomatic vines.

DA analysis identified members of the wood fungal microbiome that were specifically associated with asymptomatic or symptomatic vines. Considering the whole dataset, *Acremonium alternatum*, one of the most dominant ASV in the dataset overall, and *K. variispora*, were identified as indicators of symptomatic plants. The former is a common saprotrophic endophyte, previously isolated as grapevine endophyte [5, 58], not exhibiting plant pathogenic traits. *K. variispora* has been characterized as a common wood fungus of vine, that causes irregular discoloration of vascular tissues, necrotic spots, carcinomas, and grapevine decline [20]. In Greece, this fungus has been described as a causal agent of wood necrosis and fruit rot on apples [59]. The fungus produces two polyketide massarilactones that were phytotoxic, reproducing grapevine decline symptoms on plant leaves [18]. Conversely, we identified an *Alternaria* ASV that was consistently associated with asymptomatic plants regardless of cultivar/viticultural zones. Fungi of this genus are common members of the cultivable grapevine wood microbiome [5, 25]. Their consistent association with asymptomatic vines suggests a role in suppression of GTDs, further reinforced by the negative co-occurrence network pattern of *Alternaria* with hallmark GTD fungal genera like *Phaemomoniella* and *Phaeoacromonium*. In line with this Del Frari et al. [58] showed that a consortium composed of four endophytic fungi, including *A. alternata*, isolated from asymptomatic vines was able to decrease *P. chlamydospora* infection *in planta*. When looking at each cultivar separately, we noted different GTD-associated pathogens to be consistently present in symptomatic plants like *Fomitiporia* spp., in Agiorgitiko, *P. chlamydospora*, *K. variispora* and *Diaporthe* spp., in Vidiano. Bruez et al. [24] identified *Fomitiporia* as the single member of the wood microbiome that was consistently more abundant in white rot wood tissues of visibly healthy and diseased plants. They suggested that it is the key for the onset of the white rot syndrome collaborating with primary vascular tissue colonizers like *P. chlamydospora*, and *Phaeoacremonium* spp.; both of these fungi were prevalent in our study, and the latter dominated in the symptomatic Xinomavro vines. The key role of *Fomitiporia*, along with the two trachemycotic fungi (*P. chlamydospore* and *Phaeoacremonium* sp.) and other confounding factors like climatic conditions and vineyard age, in leaf symptoms manifestation was recently highlighted in two comprehensive reviews by Morreti et al. [60] and Del Frari et al. [19].

A range of wood endophytic fungi previously reported as members of the wood grapevine microbiome like *L. cynaroidis* [24] and *M. restricta* [25, 58], but also fungi that have been reported as wood pathogens involved in GTDs like *Eutypa* spp., and *S. vitis* in Xinomavro [61, 62], were associated with asymptomatic vines. The consistent presence of these pathogens in asymptomatic plants is not surprising, and previous studies have also reported the common occurrence of GTD-related pathogens in asymptomatic plants [3, 5].

A Random Forest supervised learning model was used to identify members of the fungal wood microbiome that could be used as predictors of GTD symptomatic samples. This approach pointed to *P. chlamydospora* and *K. variispora*, both being members of the GTD-related pathobiome, along with *A. alternatum* and *Cladosporium* spp., not previously reported as wood pathogens in grapevine, as highly accurate predictors of symptomatic plants. Wood microbiome analysis of grapevines based on these predictors could be used as an early warning for the appearance of GTDs. Further testing with a wider dataset will verify the utility of such an approach in controlling the spread of GTDs in global vineyards.

### Bacterial microbiome

The bacterial wood microbiome was overly dominated by *Proteobacteria*, and mostly α- and γ-proteobacteria, followed by *Actinobacteria* and *Bacteroidetes,* in line with previous studies [24, 31, 32]. Amongst those phyla, members of the families *Sphingomonadaceae* and *Bacillaceae* were the most prevalent. Previous culture-dependent and culture-independent efforts to record the grapevine wood bacteriome have identified *Bacillaceae* and *Sphingomonadaceae* amongst the most abundant members of this community [24, 63]. The bacterial community shared similar assembly mechanisms with the fungal community, and it was strongly driven by biogeography/cultivar as a lumped factor, unlike GTD status which did not show a high discriminatory power. Previous studies looking at the vine microbiome suggested that biogeography was the most important factor shaping bacterial communities in berries [29], vineyard soil [64, 65] and the bark of grapevines [66]. The few studies that have explored the composition of the bacterial wood microbiome identified limited differences between symptomatic and asymptomatic vines [24, 32], in accordance with our findings. However, in line with the fungal microbiome, we noted cultivar- / viticultural zone-specific differentiation between symptomatic and asymptomatic vines in the cultivar Xinomavro. It is not clear what may be the driving factors for the distinct assemblages of both fungal and bacterial communities in the Xinomavro cultivar compared with the other two, more susceptible to GTD cultivars. On-going analysis of the wood microbiome of this cultivar in symptomatic and asymptomatic vines across viticultural zones will shed further light into this hypothesis.

When looking for bacterial drivers of the distinction between asymptomatic and symptomatic plants, we identified a strong enrichment of *Bacillus* (and widely of members of the family *Bacillaceae*) and *Streptomyces* in the former. This enrichment was particularly prominent in the cultivars Xinomavro and Agiorgitiko, while in Vidiano we noted, in addition to *Bacillus*, an enrichment of *Corynebacterium*. Bruez et al. [32] identified *Bacillus* spp. as major components of the wood bacterial community in vines; however, they did not observe a significant differentiation in their abundance between symptomatic and asymptomatic plants. This was further confirmed in a follow up study on the same vineyard using high throughput sequencing [24]. In the same study *Streptomyces* showed a significantly higher abundance in asymptomatic plants (7% vs < 0.1%), in line with our findings. The consistent prevalence of *Bacillus* and *Streptomyces* in the asymptomatic plants and their negative co-occurrence patterns with the GTD pathogens *Phaeoacremonium, Phaeomoniella* and *Seimatosporium* in asymptomatic vines could be associated with the capacity of members of these bacterial genera to act as biocontrol agents and/or plant growth promoters in several plants including grapevines [67, 68]. Indeed Rezgui et al. [69] and Haidar et al. [70] reported a high suppressive activity of endophytic *Bacillus* strains isolated from vines against GTD-related pathogens like *P. chlamydospora, Lasidiodiplodia pseudotheobromae, Neofusicoccum parvum* and *Schizophyllum commune*, acting through the production of VOCs, antibiotics or by inducing systematic resistance in the plant host. Alvarez-Perez et al. [71] isolated an endophytic *Streptomyces* from asymptomatic grapevines that suppressed *P. chlamydospora* and *Phaeoacremonium minimum in planta* and in vitro. Similarly, Trotel-Aziz et al. [72] showed that a *Bacillus subtilis* strain, isolated from grapevine rhizosphere, was able to attenuate Botryosphaeria dieback by triggering host immune responses and detoxifying phytotoxins produced by *N. parvum*, mechanisms further verified by genomic analysis of the studied strain [73]. On-going high-throughput isolation effort will explore this rich endophytic bacterial community of asymptomatic plants for novel potential bacterial control agents against GTDs.

## Conclusions

We studied the wood microbiome of grapevines from three Greek cultivars located in three geographically distinct viticultural zones following a systematic sampling approach distinguishing between GTD-symptomatic and asymptomatic plants. Geographical location and/or cultivar were the main determinants of both fungal and bacterial microbiome, unlike the GTD status of grapevines that differentiated between asymptomatic and symptomatic plants only for the most resistant to GTDs cultivar Xinomavro, suggesting complex interactions between cultivar/biogeography and the wood fungal pathobiome. *P. chlamydospora*, *K. variispora*, *Fomitiporia* spp., *Diaporthe* spp., all previously proposed as causal agents of GTDs, along with *Acremonium* sp. (non GTD-linked fungus) were consistently associated with symptomatic vines in a cultivar-dependent manner. In addition, the first two of these pathogens, along with *Cladosporium* spp. and *A. alternatum* were identified, via Random Forest analysis, as highly accurate predictors of symptomatic vines. The bacterial microbiome in asymptomatic plants was dominated by *Bacillus* and *Streptomyces,* which showed a negative co-occurrence pattern with *Phaeomoniella, Phaeoacremonium* and *Seimatosporium.* This suggested a role of in the suppression of GTDs, a hypothesis which warrants verification through isolation and phenotypic characterization of these bacteria. Overall, we provide evidence that GTD symptomatic plants support a wood fungal microbiome showing cultivar and/or biogeography-dependent patterns. Specific members of this fungal wood microbiome could be used as a proxy to distinguish between healthy and diseased vines. Further tests with similar datasets from different viticultural regions or at large geographical scale would verify the universal utility of this approach. Potential interactions between the bacterial and fungal wood microbiome in GTD-free vines should be further pursued in the quest for discovery of novel grapevine-accustomed biocontrol agents.

## Supplementary Information

**Additional file 1**. **Figure S1.** A map of Greece showing the sampling sites (Amyntaio, Nemea and Crete). Vineyards of Xinomavro, Agiorgitiko and Vidiano cultivars were sampled in Amyntaio, Nemea and Crete regions, respectively. **Figure S2.** Rarefaction curves for the wood samples analyzed for their fungal (a) and bacterial microbiome (b). **Figure S3.** Stacked bar plots showing the composition of the fungal community (at the class taxonomic level) in wood samples collected from GTD asymptomatic and symptomatic vines of the cultivars Agiorgitiko (a), Xinomavro (b), Vidiano (c) each located in a distinct viticultural zone in Greece. **Figure S4.** Fungal ASVs phylogenetically assigned to taxa previously identified as causal agents of GTDs that showed differential abundance in samples collected from symptomatic and asymptomatic vines. Data are presented collectively for all geographic locations/cultivars (a) and for each variety separately (b) Agiorgitiko (c) Xinomavro (d) Vidiano. ASV014: *Phaeomoniella chlamydospora*, ASV068: *Kalmusia variispora*, ASV13: *Seimatosporium vitis*, ASV52: *Fomitiporia* spp., ASV338: *Seimatosporium vitis*, ASV005: *Phaeomoniella chlamydospora*, ASV008: *Phaeoacremonium iranianum*, ASV75: *Neosetophoma* spp., ASV159: *Neosetophoma* spp., ASV074: *Diaporthe* spp., (Signif. Differences: ‘***’ 0.001 ‘**’ 0.01 ‘*’ 0.05). **Figure S5.** Stacked bar plots showing the composition of the bacterial community (at the phylum taxonomic level and for the class Proteobacteria) in wood samples collected from GTP asymptomatic and symptomatic vines of the cultivars Agiorgitiko (a), Xinomavro (b), Vidiano (c) each located in a distinct viticultural zone in Greece. **Figure S6.** Out of bag (OOB) error rates of the Random Forest model parameter states next to the overall model error rates (7.94% shown with the black line) for each one of the 1000 model trees. The confusion matrix is also provided on the plot. **Table S1:** A list of the wood samples analyzed with all relevant information regarding vine cultivar and viticultural zone, GTD symptoms presence or absence, plant part sampled, vineyard code, plant code and geographic location. **Table S2.** Primers used for amplicon sequencing analysis. B000X-515f and FI000X-ITS4r are indexed primers used in the second amplification step, which are composed of the sequence of the universal primers 515f (bacteria) and ITS4r (fungi) (bold), the indexes used for samples barcoding (underlined) and a TT (linker) sequence at the 5' end of each primer. **Table S3.** PCR reagents and thermocycling conditions used for amplicon sequencing analysis. **Table S4.** PERMANOVA analysis of the fungal and bacterial wood microbiome (Signif. codes: 0.001 ‘***’ 0.01 ‘**’ 0.05 ‘*’). **Table S5.** ASVs detected in the wood microbiome of the vines studied that are considered as putative pathogens involved in GTDs. Each ASV was phylogenetically assigned to the closest verified taxonomic level (genus or species).

## Data Availability

The datasets generated during and/or analysed during the current study are available from the corresponding author on reasonable request.

## Abbreviations

GTDs: Grapevine trunk diseases
ASVs: Amplicon sequence variants
CTAB: Cetyltetramethyl ammonium bromide
NMDS: Non-metric multidimensional scaling
PERMANOVA: Permutational multivariate analysis of variance
DA: Differential abundance
OOB: Out of bag
