# Correction to: Grapevine wood microbiome analysis identifies key fungal pathogens and potential interactions with the bacterial community implicated in grapevine trunk disease appearance

**DOI:** 10.1186/s40793-022-00405-5

**Published:** 2022-03-07

**Authors:** Fotios Bekris, Sotirios Vasileiadis, Elena Papadopoulou, Anastasios Samaras, Stefanos Testempasis, Danae Gkizi, Georgia Tavlaki, Aliki Tzima, Epaminondas Paplomatas, Emmanuel Markakis, George Karaoglanidis, Kalliope K. Papadopoulou, Dimitrios G. Karpouzas

**Affiliations:** 1 Laboratory of Plant and Environmental Biotechnology, Viopolis, Department of Biochemistry and Biotechnology, University of Thessaly, 41500 Larissa, Greece; 2 Plant Pathology Laboratory, Faculty of Agriculture, Aristotle University of Thessaloniki, Thessaloniki, Greece; 3 Laboratory of Plant Pathology, Agricultural University of Athens, Iera Odos 75, 11855 Athens, Greece; 4 Laboratory of Mycology, Department of Viticulture, Vegetable Crops, Floriculture and Plant Protection, Institute of Olive Tree, Subtropical Crops and Viticulture, Hellenic Agricultural Organization DIMITRA, 32A Kastorias Street, Mesa Katsabas, 71307 Heraklion, Crete Greece

## Correction to: Environmental Microbiome (2021) 16:23 10.1186/s40793-021-00390-1

Following publication of the article [1], the authors flagged that each author's first and last names had been erroneously swapped in the author list.

The author list has been corrected in the published article and the correct author list may be found in this erratum.

