# Supplementary material for: Grapevine wood microbiome analysis identifies key fungal pathogens and potential interactions with the bacterial community implicated in grapevine trunk disease appearance
Source: Environ Microbiome. 2021 Dec 4;16:23. doi: 10.1186/s40793-021-00390-1 (PMC8642934; doi:10.1186/s40793-021-00390-1)
Supplement: Supplementary file 1 — Additional file 1. Figure S1. A map of Greece showing the sampling sites (Amyntaio, Nemea and Crete). Vineyards of Xinomavro, Agiorgitiko and Vidiano cultivars were sampled in Amyntaio, Nemea and Crete regions, respectively. Figure S2. Rarefaction curves for the wood samples analyzed for their fungal (a) and bacterial microbiome (b). Figure S3. Stacked bar plots showing the composition of the fungal community (at the class taxonomic level) in wood samples collected from GTD asymptomatic and symptomatic vines of the cultivars Agiorgitiko (a), Xinomavro (b), Vidiano (c) each located in a distinct viticultural zone in Greece. Figure S4. Fungal ASVs phylogenetically assigned to taxa previously identified as causal agents of GTDs that showed differential abundance in samples collected from symptomatic and asymptomatic vines. Data are presented collectively for all geographic locations/cultivars (a) and for each variety separately (b) Agiorgitiko (c) Xinomavro (d) Vidiano. ASV014: Phaeomoniella chlamydospora, ASV068: Kalmusia variispora, ASV13: Seimatosporium vitis, ASV52: Fomitiporia spp., ASV338: Seimatosporium vitis, ASV005: Phaeomoniella chlamydospora, ASV008: Phaeoacremonium iranianum, ASV75: Neosetophoma spp., ASV159: Neosetophoma spp., ASV074: Diaporthe spp., (Signif. Differences: ‘***’ 0.001 ‘**’ 0.01 ‘*’ 0.05). Figure S5. Stacked bar plots showing the composition of the bacterial community (at the phylum taxonomic level and for the class Proteobacteria) in wood samples collected from GTP asymptomatic and symptomatic vines of the cultivars Agiorgitiko (a), Xinomavro (b), Vidiano (c) each located in a distinct viticultural zone in Greece. Figure S6. Out of bag (OOB) error rates of the Random Forest model parameter states next to the overall model error rates (7.94% shown with the black line) for each one of the 1000 model trees. The confusion matrix is also provided on the plot. Table S1: A list of the wood samples analyzed with all relevant information regarding v [file 40793_2021_390_MOESM1_ESM.docx]

**Supplementary Data**

**Grapevine wood microbiome analysis identifies key fungal pathogens and potential interactions with the bacterial community implicated in grapevine trunk disease appearance**

Bekris F.,^1^ Vasileiadis S.,^1^ Papadopoulou E.,^1^ Samaras A.,^2^ Testempasis S.,^2^ Gkizi D^3^, Tavlaki, G.,^4^ Tzima A.,^3^ Paplomatas E.,^3^ Markakis E.,^4^ Karaoglanidis G.,^2^ Papadopoulou K.K.,^1^ Karpouzas D.G.,^1*^

*^1^ University of Thessaly, Department of Biochemistry and Biotechnology, Laboratory of Plant and Environmental Biotechnology, Viopolis – 41500 Larissa, Greece*

*^2^ Plant Pathology Laboratory, Faculty of Agriculture, Aristotle University of Thessaloniki, Thessaloniki, Greece*

*^3^ Laboratory of Plant Pathology, Agricultural University of Athens, Iera Odos 75, 11855 Athens, Greece*

*^4^ Laboratory of Mycology, Department of Viticulture, Vegetable Crops, Floriculture and Plant Protection, Institute of Olive Tree, Subtropical Crops and Viticulture, Hellenic Agricultural Organization DIMITRA, 32^A^ Kastorias street, Mesa Katsabas 71307, Heraklion, Crete, Greece*

**Corresponding author^+^**

Dimitrios Karpouzas

Tel. +302410565294, Fax: +302410565290

Email. [dkarpouzas@bio.uth.gr](mailto:dkarpouzas@bio.uth.gr)

**Supplementary Figures**

**Supplementary Figure S1.** A map of Greece showing the sampling sites (Amyntaio, Nemea and Crete). Vineyards of Xinomavro, Agiorgitiko and Vidiano cultivars were sampled in Amyntaio, Nemea and Crete regions, respectively.

**Supplementary Figure S2.** Rarefaction curves for the wood samples analyzed for their fungal (a) and bacterial microbiome (b).

**Supplementary Figure S3.** Stacked bar plots showing the composition of the fungal community (at the class taxonomic level) in wood samples collected from GTD asymptomatic and symptomatic vines of the cultivars Agiorgitiko (a), Xinomavro (b), Vidiano (c) each located in a distinct viticultural zone in Greece.

**Supplementary Figure S4.** Fungal ASVs phylogenetically assigned to taxa previously identified as causal agents of GTDs that showed differential abundance in samples collected from symptomatic and asymptomatic vines. Data are presented collectively for all geographic locations/cultivars (a) and for each variety separately (b) Agiorgitiko (c) Xinomavro (d) Vidiano. ASV014: *Phaeomoniella chlamydospora*, ASV068: *Kalmusia variispora*, ASV13: *Seimatosporium vitis*, ASV52: *Fomitiporia* spp*.*, ASV338: *Seimatosporium vitis*, ASV005: *Phaeomoniella chlamydospora*, ASV008: *Phaeoacremonium iranianum*, ASV75: *Neosetophoma* spp*.*, ASV159: *Neosetophoma* spp*.*, ASV074: *Diaporthe* spp., (Signif. Differences: ‘***’ 0.001 ‘**’ 0.01 ‘*’ 0.05).

**Supplementary Figure S5**. Stacked bar plots showing the composition of the bacterial community (at the phylum taxonomic level and for the class Proteobacteria) in wood samples collected from GTP asymptomatic and symptomatic vines of the cultivars Agiorgitiko (a), Xinomavro (b), Vidiano (c) each located in a distinct viticultural zone in Greece

**Supplementary Figure S6.** Network analysis of the fungal wood microbiome (a,b) and of the GTD-associated fungal genera with the wood bacterial microbiome (c,d) in symptomatic (a,c) and asymptomatic (b,d) vines, regardless of cultivar/viticultural zones. Regarding the wood fungal and bacterial microbiome analysis, only fungal and bacterial genera which showed a relative abundance >1% in 10% of the samples analyzed were considered in the analysis. Blue and green bubbles designate fungal and bacterial genera respectively, while the size of each bubble indicates the relative abundance of each microbial genera. Green and red links signify significant positive and negative co-occurrence patterns between the linked microorganisms while the width of the line is a measure of the level of the co-occurrence correlation between the linked microorganisms (the higher the width of the line the higher the correlation between the co-occurrence of the microorganisms).

**Supplementary Figure S7**. Out of bag (OOB) error rates of the Random Forest model parameter states next to the overall model error rates (7.94% shown with the black line) for each one of the 1000 model trees. The confusion matrix is also provided on the plot.

**Supplementary Tables**

**Supplementary Table S1:** A list of the wood samples analyzed with all relevant information regarding vine cultivar and viticultural zone, GTD symptoms presence or absence, plant part sampled, vineyard code, plant code and geographic location.

**Supplementary Table S2.** Primers used for amplicon sequencing analysis. B000X-515f and FI000X-ITS4r are indexed primers used in the second amplification step, which are composed of the sequence of the universal primers 515f (bacteria) and ITS4r (fungi) (bold), the indexes used for samples barcoding (underlined) and a TT (linker) sequence at the 5' end of each primer.

**Supplementary Table S3.** PCR reagents and thermocycling conditions used for amplicon sequencing analysis.

**Supplementary Table S4.** PERMANOVA analysis of the fungal and bacterial wood microbiome (Signif. codes: 0.001 ‘***’ 0.01 ‘**’ 0.05 ‘*’).

**Supplementary Table S5.** ASVs detected in the wood microbiome of the vines studied that are considered as putative pathogens involved in GTDs. Each ASV was phylogenetically assigned to the closest verified taxonomic level (genus or species)

**
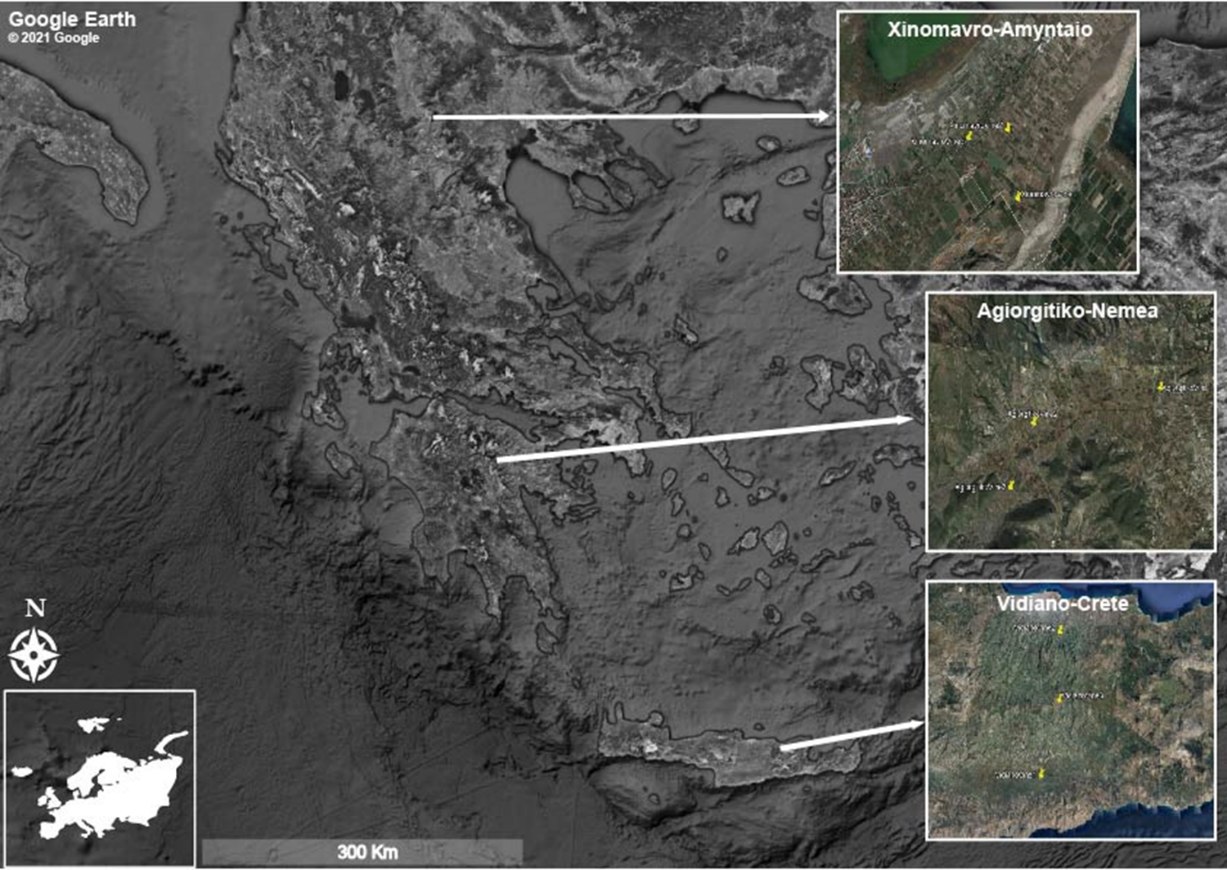
**

**Supplementary Figure S1.** A map of Greece showing the sampling sites (Amyntaio, Nemea and Crete). Vineyards of Xinomavro, Agiorgitiko and Vidiano cultivars were sampled in Amyntaio, Nemea and Crete regions, respectively.

**
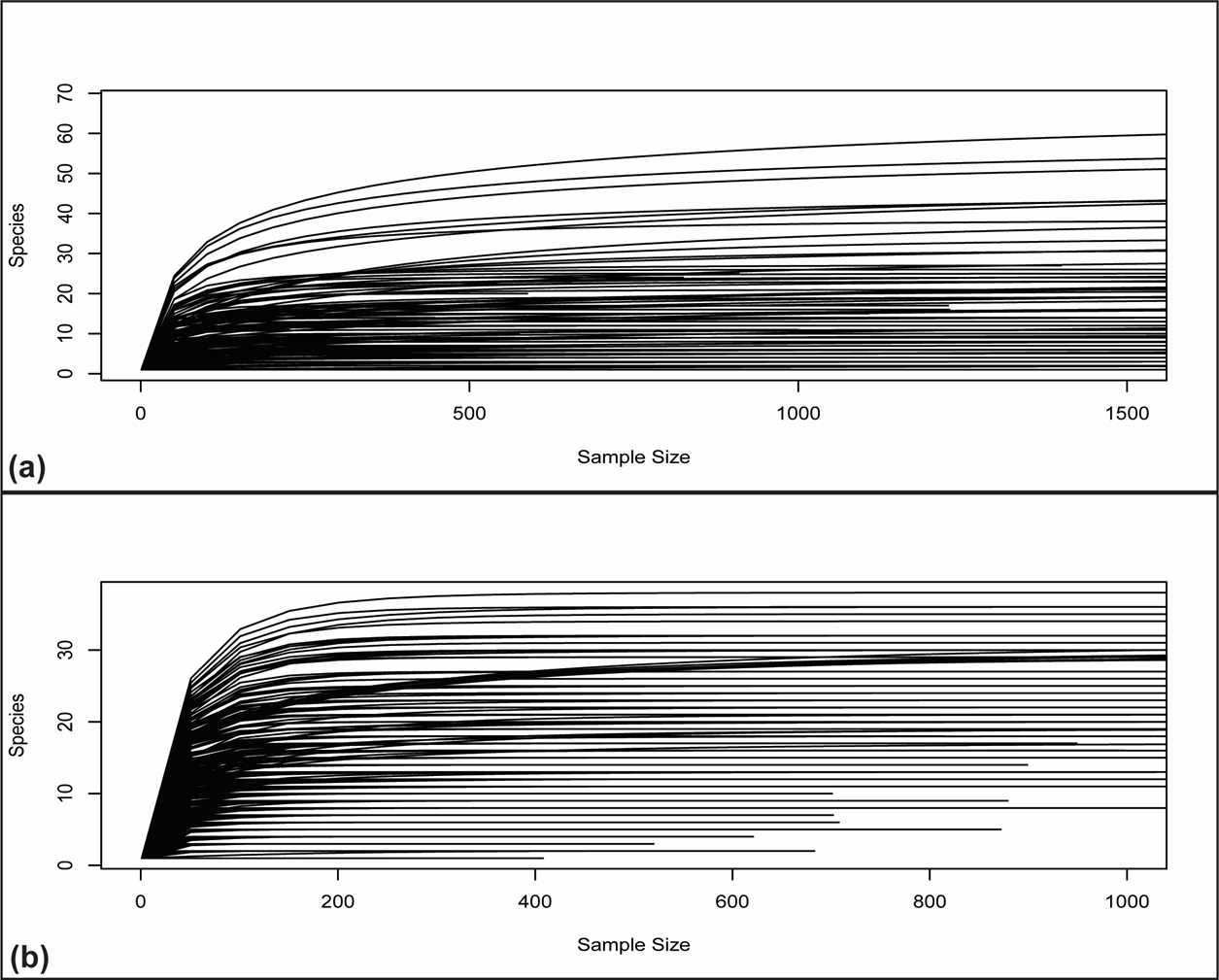
**

**Supplementary Figure S2.** Rarefaction curves for the wood samples analyzed for their fungal (a) and bacterial microbiome (b).


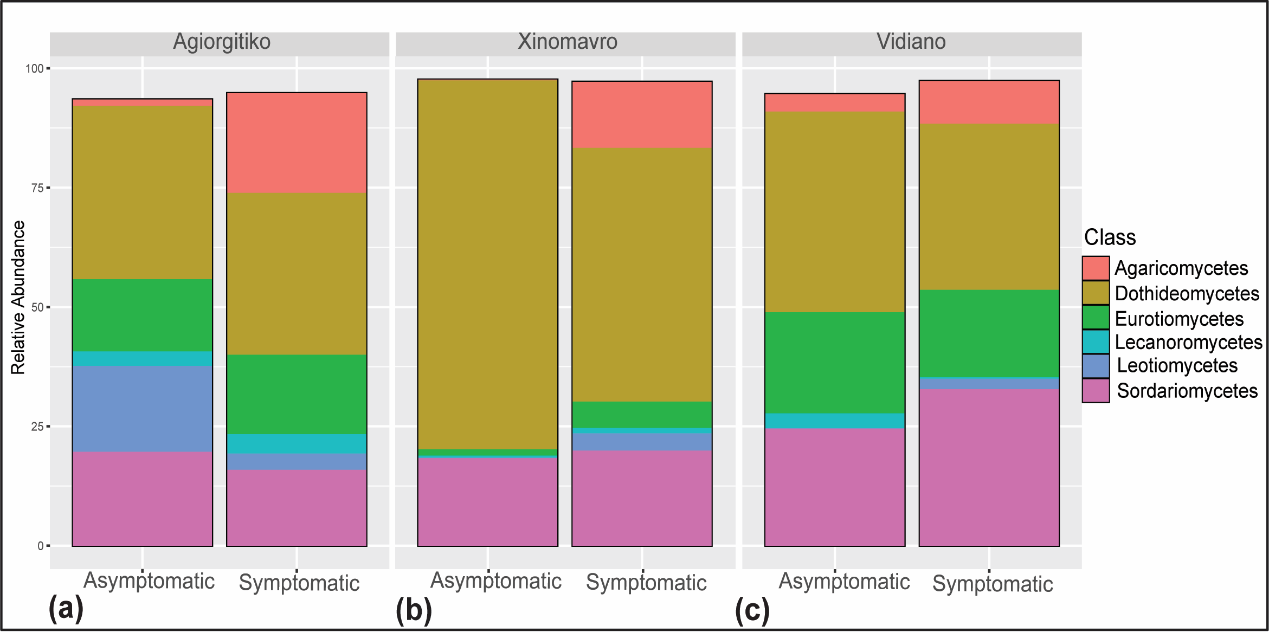


**Supplementary Figure S3.** Stacked bar plots showing the composition of the fungal community (at the class taxonomic level) in wood samples collected from GTD asymptomatic and symptomatic vines of the cultivars Agiorgitiko (a), Xinomavro (b), Vidiano (c) each located in a distinct viticultural zone in Greece.


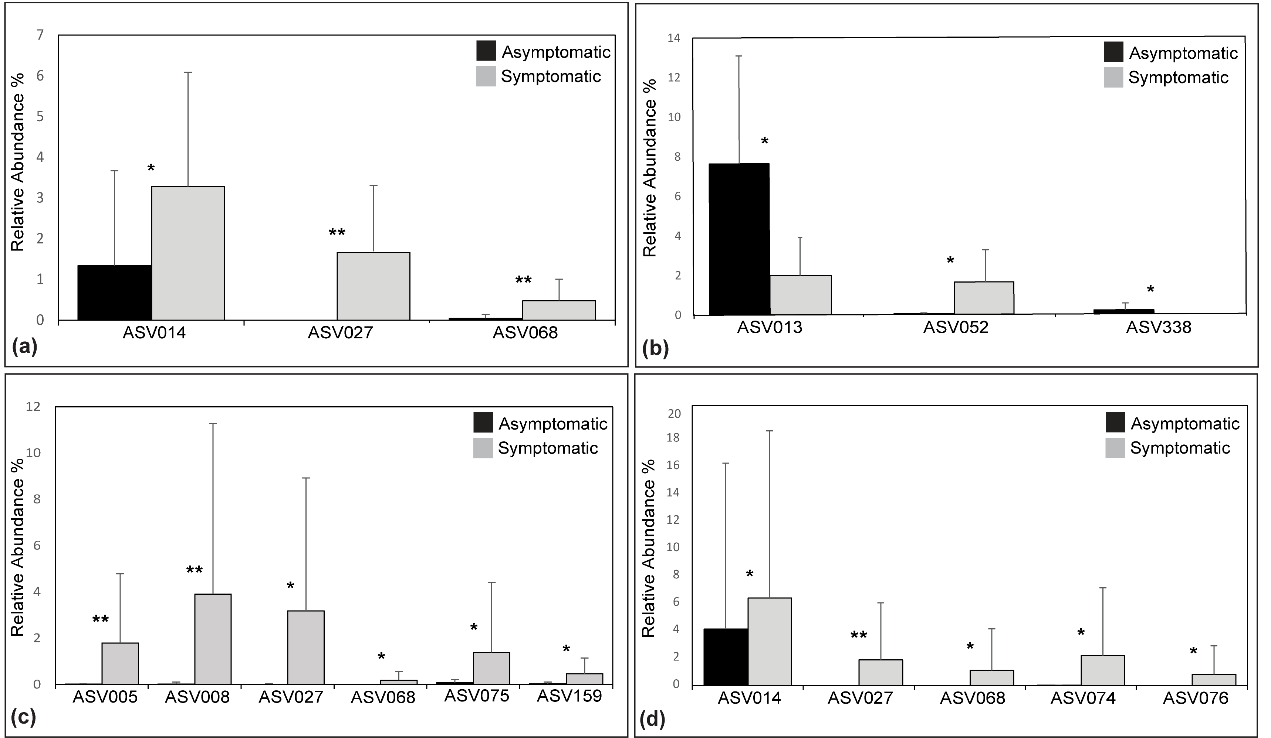


**Supplementary Figure S4.** Fungal ASVs phylogenetically assigned to taxa previously identified as causal agents of GTDs that showed differential abundance in samples collected from symptomatic and asymptomatic vines. Data are presented collectively for all geographic locations/cultivars (a) and for each variety separately (b) Agiorgitiko (c) Xinomavro (d) Vidiano. ASV014: *Phaeomoniella chlamydospora*, ASV068: *Kalmusia variispora*, ASV13: *Seimatosporium vitis*, ASV52: *Fomitiporia* spp*.*, ASV338: *Seimatosporium vitis*, ASV005: *Phaeomoniella chlamydospora*, ASV008: *Phaeoacremonium iranianum*, ASV75: *Neosetophoma* spp*.*, ASV159: *Neosetophoma* spp*.*, ASV074: *Diaporthe* spp., (Signif. Differences: ‘***’ 0.001 ‘**’ 0.01 ‘*’ 0.05).

**
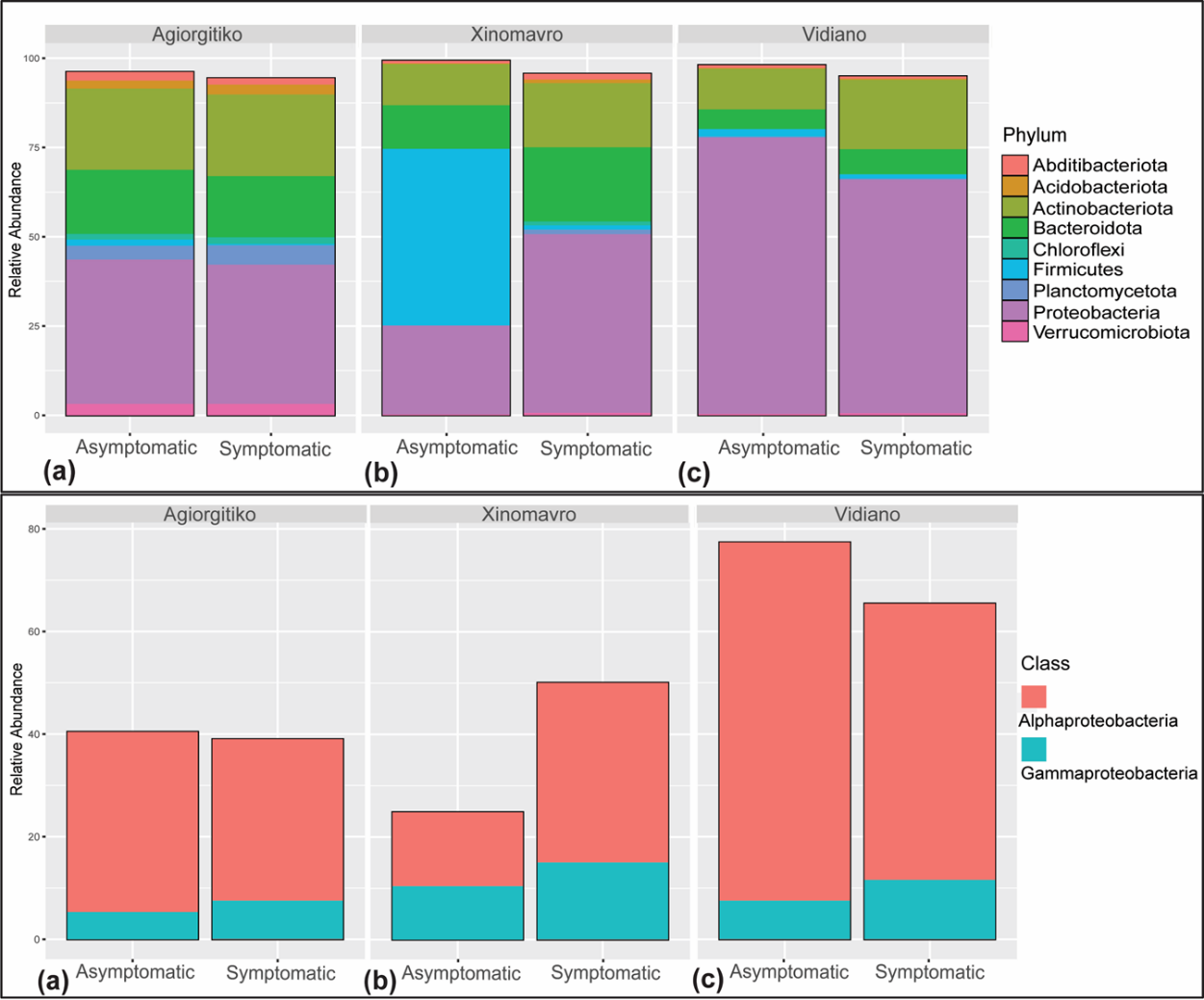
**

**Supplementary Figure S5**. Stacked bar plots showing the composition of the bacterial community (at phylum taxonomic level and for the class Proteobacteria) in wood samples collected from GTP asymptomatic and symptomatic vines of the cultivars Agiorgitiko (a), Xinomavro (b), Vidiano (c) each located in a distinct viticultural zone in Greece.

| 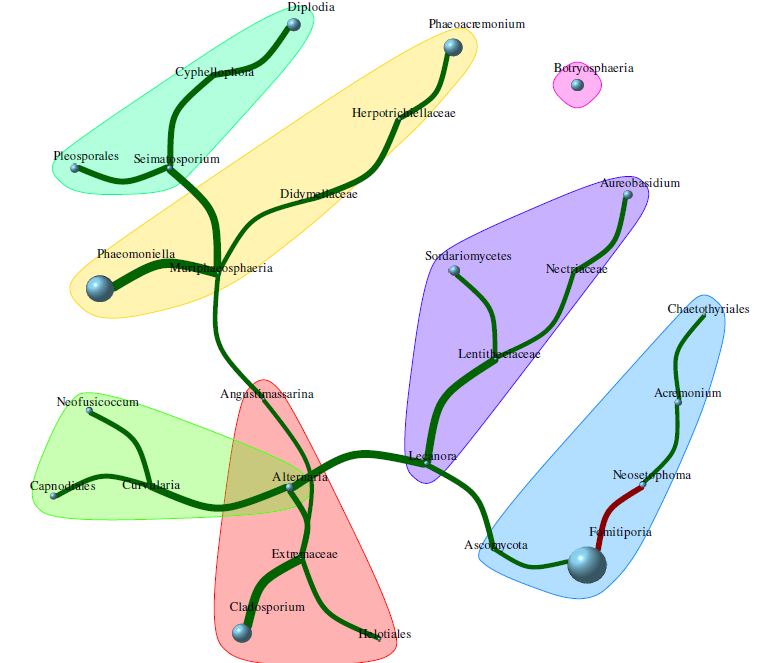  **(a)** | 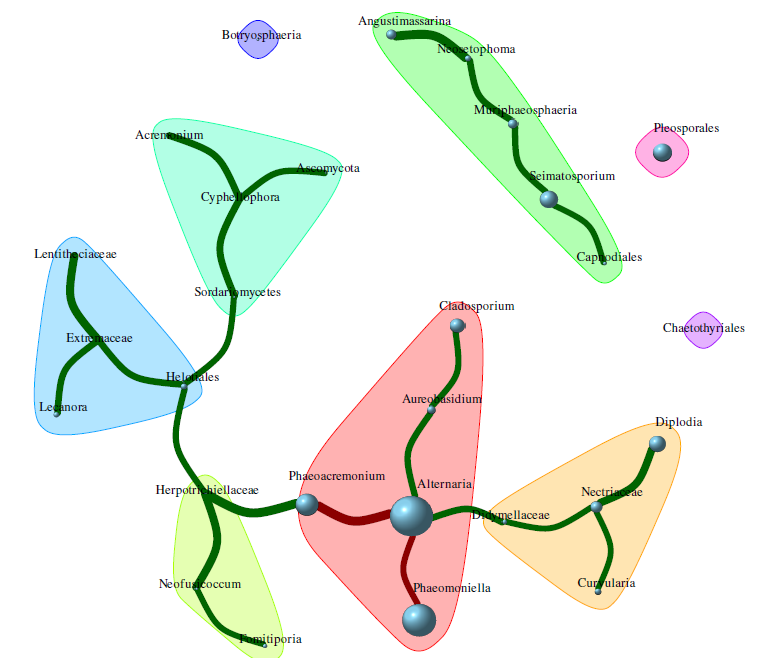  **(b)** |
| --- | --- |
| 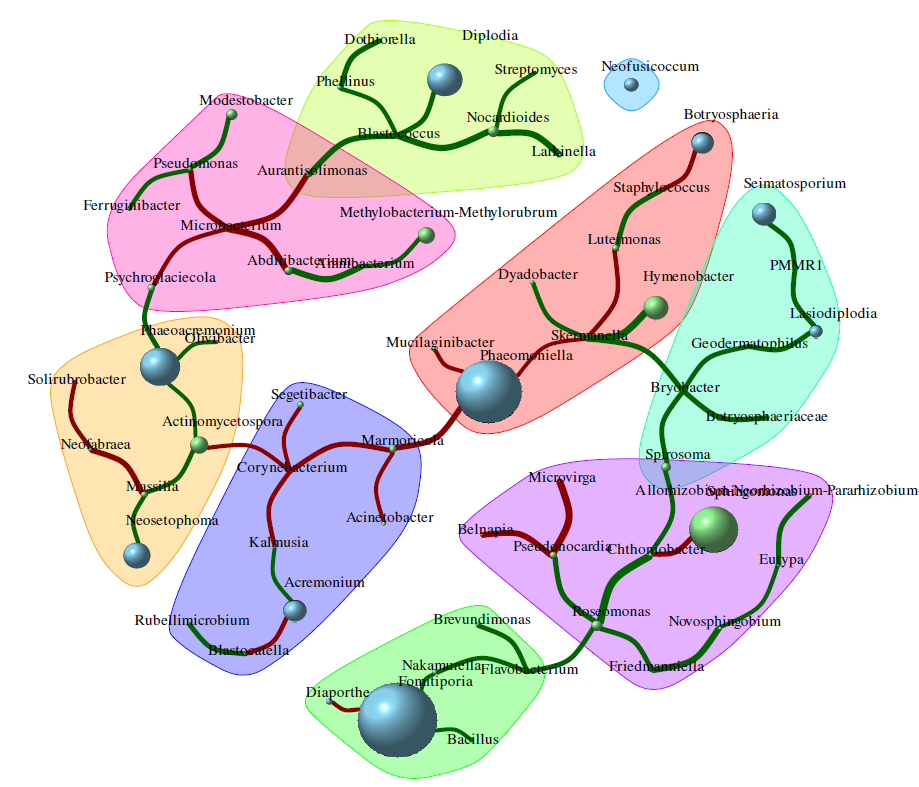  **(c)** | 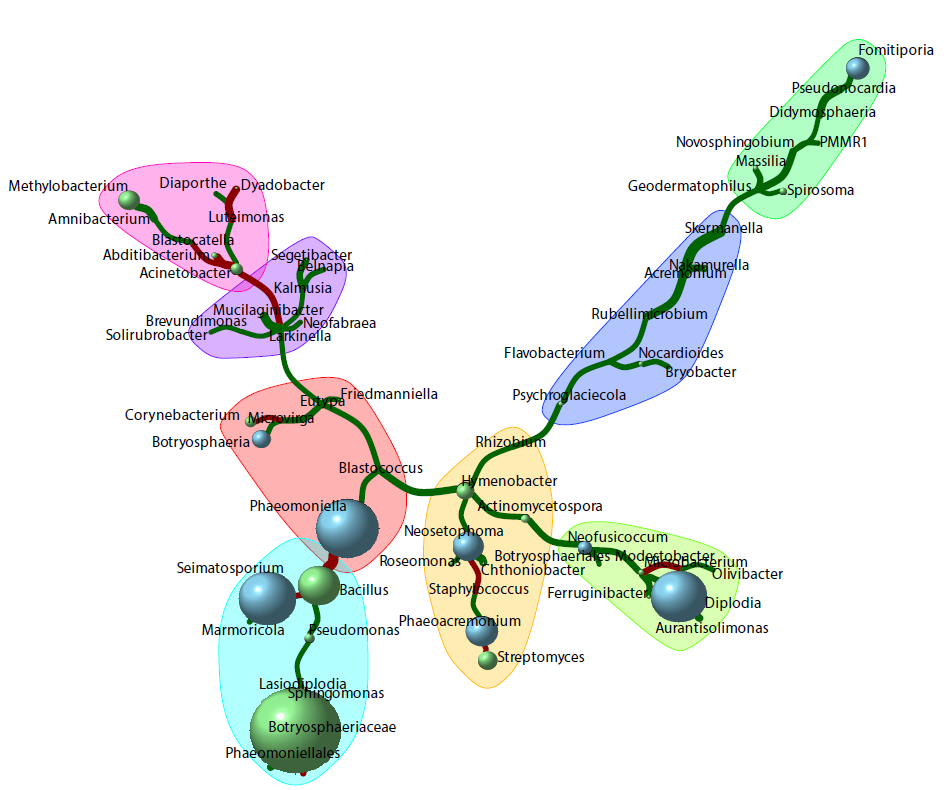  **(d)** |

**Supplementary Figure S6.** Network analysis of the fungal wood microbiome (a,b) and of the GTD-associated fungal genera with the wood bacterial microbiome (c,d) in symptomatic (a,c) and asymptomatic (b,d) vines, regardless of cultivar/viticultural zones. Regarding the wood fungal and bacterial microbiome analysis, only fungal and bacterial genera which showed a relative abundance >1% in 10% of the samples analyzed were considered in the analysis. Blue and green bubbles designate fungal and bacterial genera respectively, while the size of each bubble indicates the relative abundance of each microbial genera. Green and red links signify significant positive and negative co-occurrence patterns between the linked microorganisms while the width of the line is a measure of the level of the co-occurrence correlation between the linked microorganisms (the higher the width of the line the higher the correlation between the co-occurrence of the microorganisms).

**Supplementary Figure S7**. Out of bag (OOB) error rates of the Random Forest model parameter states next to the overall model error rates (7.94% shown with the black line) for each one of the 1000 model trees. The confusion matrix is also provided on the plot.

**Supplementary Table S1:** A list of the wood samples analyzed with all relevant information regarding vine cultivar and viticultural zone, GTDs symptoms presence or absence, plant part sampled, vineyard code, plant code and geographic location.

| **Sample Νο** | **Cultivar – Viticultural Zone** | **GTD Condition** | **Plant part** | **Vineyard Code Number** | **Plant Code Number** | **Geographic location** |
| --- | --- | --- | --- | --- | --- | --- |
| 1 | Agiorgitiko-Nemea | Symptomatic | Cordon | No3I | No1 | Peloponnese |
| 2 | Agiorgitiko-Nemea | Symptomatic | Trunk | No3I | No1 | Peloponnese |
| 3 | Agiorgitiko-Nemea | Symptomatic | Trunk | No3I | No2 | Peloponnese |
| 4 | Agiorgitiko-Nemea | Symptomatic | Cordon | No3I | No3 | Peloponnese |
| 5 | Agiorgitiko-Nemea | Symptomatic | Cordon | No1G | No1 | Peloponnese |
| 6 | Agiorgitiko-Nemea | Symptomatic | Trunk | No1G | No1 | Peloponnese |
| 7 | Agiorgitiko-Nemea | Symptomatic | Cordon | No1G | No2 | Peloponnese |
| 8 | Agiorgitiko-Nemea | Symptomatic | Trunk | No1G | No2 | Peloponnese |
| 9 | Agiorgitiko-Nemea | Symptomatic | Cordon | No1G | No3 | Peloponnese |
| 10 | Agiorgitiko-Nemea | Symptomatic | Trunk | No1G | No3 | Peloponnese |
| 11 | Agiorgitiko-Nemea | Symptomatic | Cordon | No2H | No1 | Peloponnese |
| 12 | Agiorgitiko-Nemea | Symptomatic | Trunk | No2H | No1 | Peloponnese |
| 13 | Agiorgitiko-Nemea | Symptomatic | Cordon | No2H | No2 | Peloponnese |
| 14 | Agiorgitiko-Nemea | Symptomatic | Trunk | No2H | No2 | Peloponnese |
| 15 | Agiorgitiko-Nemea | Symptomatic | Cordon | No2H | No3 | Peloponnese |
| 16 | Agiorgitiko-Nemea | Symptomatic | Trunk | No2H | No3 | Peloponnese |
| 17 | Agiorgitiko-Nemea | Asymptomatic | Trunk | No1G | No3 | Peloponnese |
| 18 | Agiorgitiko-Nemea | Asymptomatic | Trunk | No2H | No2 | Peloponnese |
| 19 | Agiorgitiko-Nemea | Asymptomatic | Trunk | No2H | No3 | Peloponnese |
| 20 | Agiorgitiko-Nemea | Asymptomatic | Trunk | No3I | No1 | Peloponnese |
| 21 | Xinomavro-Amyntaio | Symptomatic | Trunk | No4E | No1 | North-West Greece |
| 22 | Xinomavro-Amyntaio | Symptomatic | Trunk | No4E | No3 | North-West Greece |
| 23 | Xinomavro-Amyntaio | Symptomatic | Trunk | No4E | No2 | North-West Greece |
| 24 | Xinomavro-Amyntaio | Symptomatic | Cordon | No4E | No2 | North-West Greece |
| 25 | Xinomavro-Amyntaio | Symptomatic | Cordon | No4E | No1 | North-West Greece |
| 26 | Xinomavro-Amyntaio | Symptomatic | Trunk | No4E | No3 | North-West Greece |
| 27 | Xinomavro-Amyntaio | Symptomatic | Trunk | No5D | No1 | North-West Greece |
| 28 | Xinomavro-Amyntaio | Symptomatic | Cordon | No5D | No1 | North-West Greece |
| 29 | Xinomavro-Amyntaio | Symptomatic | Trunk | No5D | No2 | North-West Greece |
| 30 | Xinomavro-Amyntaio | Symptomatic | Trunk | No5D | No3 | North-West Greece |
| 31 | Xinomavro-Amyntaio | Symptomatic | Cordon | No5D | No2 | North-West Greece |
| 32 | Xinomavro-Amyntaio | Symptomatic | Trunk | No5D | No4 | North-West Greece |
| 33 | Xinomavro-Amyntaio | Symptomatic | Cordon | No5D | No3 | North-West Greece |
| 34 | Xinomavro-Amyntaio | Symptomatic | Cordon | No5D | No4 | North-West Greece |
| 35 | Xinomavro-Amyntaio | Symptomatic | Trunk | No7F | No2 | North-West Greece |
| 36 | Xinomavro-Amyntaio | Symptomatic | Trunk | No7F | No3 | North-West Greece |
| 37 | Xinomavro-Amyntaio | Symptomatic | Cordon | No7F | No1 | North-West Greece |
| 38 | Xinomavro-Amyntaio | Symptomatic | Trunk | No7F | No1 | North-West Greece |
| 39 | Xinomavro-Amyntaio | Symptomatic | Cordon | No7F | No3 | North-West Greece |
| 40 | Xinomavro-Amyntaio | Asymptomatic | Trunk | No4E | No1 | North-West Greece |
| 41 | Xinomavro-Amyntaio | Asymptomatic | Cordon | No4E | No1 | North-West Greece |
| 42 | Xinomavro-Amyntaio | Asymptomatic | Trunk | No4E | No2 | North-West Greece |
| 43 | Xinomavro-Amyntaio | Asymptomatic | Cordon | No4E | No2 | North-West Greece |
| 44 | Xinomavro-Amyntaio | Asymptomatic | Trunk | No4E | No3 | North-West Greece |
| 45 | Xinomavro-Amyntaio | Asymptomatic | Cordon | No4E | No3 | North-West Greece |
| 46 | Xinomavro-Amyntaio | Asymptomatic | Trunk | No5D | No1 | North-West Greece |
| 47 | Xinomavro-Amyntaio | Asymptomatic | Cordon | No5D | No1 | North-West Greece |
| 48 | Xinomavro-Amyntaio | Asymptomatic | Cordon | No5D | No2 | North-West Greece |
| 49 | Xinomavro-Amyntaio | Asymptomatic | Trunk | No5D | No3 | North-West Greece |
| 50 | Xinomavro-Amyntaio | Asymptomatic | Cordon | No5D | No3 | North-West Greece |
| 51 | Vidiano-Crete | Symptomatic | Trunk | No2A | No1 | South Greece |
| 52 | Vidiano-Crete | Symptomatic | Cordon | No2A | No1 | South Greece |
| 53 | Vidiano-Crete | Symptomatic | Trunk | No2A | No2 | South Greece |
| 54 | Vidiano-Crete | Symptomatic | Cordon | No2A | No2 | South Greece |
| 55 | Vidiano-Crete | Symptomatic | Trunk | No2A | No3 | South Greece |
| 56 | Vidiano-Crete | Symptomatic | Cordon | No2A | No3 | South Greece |
| 57 | Vidiano-Crete | Symptomatic | Trunk | No6B | No1 | South Greece |
| 58 | Vidiano-Crete | Symptomatic | Cordon | No6B | No1 | South Greece |
| 59 | Vidiano-Crete | Symptomatic | Trunk | No6B | No2 | South Greece |
| 60 | Vidiano-Crete | Symptomatic | Cordon | No6B | No2 | South Greece |
| 61 | Vidiano-Crete | Symptomatic | Trunk | No6B | No3 | South Greece |
| 62 | Vidiano-Crete | Symptomatic | Trunk | No7C | No1 | South Greece |
| 63 | Vidiano-Crete | Symptomatic | Cordon | No7C | No1 | South Greece |
| 64 | Vidiano-Crete | Symptomatic | Trunk | No7C | No2 | South Greece |
| 65 | Vidiano-Crete | Symptomatic | Cordon | No7C | No2 | South Greece |
| 66 | Vidiano-Crete | Symptomatic | Trunk | No7C | No3 | South Greece |
| 67 | Vidiano-Crete | Symptomatic | Cordon | No7C | No3 | South Greece |
| 68 | Vidiano-Crete | Asymptomatic | Trunk | No2A | No1 | South Greece |
| 69 | Vidiano-Crete | Asymptomatic | Trunk | No6B | No1 | South Greece |
| 70 | Vidiano-Crete | Asymptomatic | Cordon | No2A | No1 | South Greece |
| 71 | Vidiano-Crete | Asymptomatic | Cordon | No6B | No1 | South Greece |
| 72 | Vidiano-Crete | Asymptomatic | Trunk | No6B | No2 | South Greece |
| 73 | Vidiano-Crete | Asymptomatic | Cordon | No6B | No2 | South Greece |
| 74 | Vidiano-Crete | Asymptomatic | Trunk | No6B | No3 | South Greece |
| 75 | Vidiano-Crete | Asymptomatic | Cordon | No6B | No3 | South Greece |
| 76 | Vidiano-Crete | Asymptomatic | Trunk | No7C | No1 | South Greece |
| 77 | Vidiano-Crete | Asymptomatic | Cordon | No7C | No1 | South Greece |
| 78 | Vidiano-Crete | Asymptomatic | Trunk | No2A | No2 | South Greece |
| 79 | Vidiano-Crete | Asymptomatic | Trunk | No7C | No2 | South Greece |
| 80 | Vidiano-Crete | Asymptomatic | Cordon | No7C | No2 | South Greece |
| 81 | Vidiano-Crete | Asymptomatic | Trunk | No7C | No3 | South Greece |
| 82 | Vidiano-Crete | Asymptomatic | Cordon | No7C | No3 | South Greece |
| 83 | Vidiano-Crete | Asymptomatic | Cordon | No2A | No2 | South Greece |
| 84 | Vidiano-Crete | Asymptomatic | Trunk | No2A | No3 | South Greece |
| 85 | Vidiano-Crete | Asymptomatic | Cordon | No2A | No3 | South Greece |

**Supplementary Table S2.** Primers used for amplicon sequencing analysis. B000X-515f and FI000X-ITS4r are indexed primers used in the second amplification step, which are composed of the sequence of the universal primers 515f (bacteria) and ITS4r (fungi) (bold), the indexes used for samples barcoding (underlined) and a TT (linker) sequence at the 5' end of each primer.

| **Kingdom** | **PCR step** | **Primers** | **Gene target** | **Amplicon size (bp)** | **Primer sequences (5'-3')** | **References** | |
| --- | --- | --- | --- | --- | --- | --- | --- |
| Bacteria | 1st | 515F  806R | 16S rRNA | ~290 | GTGCCAGCMGCCGCGGTAA  GGACTACHVHHHTWTCTAAT | Parada et al., (2016) |  |
|  | 2nd | Indexed(n=85) | B0001-515f  B0002-515f  B0003-515f  B0004-515f  B0005-515f |  | TTCTTCTTCGT**GTGYCAGCMGCCGCGGTAA**  TTCTCAATGGT**GTGYCAGCMGCCGCGGTAA**  TTCAGTTCAGT**GTGYCAGCMGCCGCGGTAA**  TTCGAATCAGT**GTGYCAGCMGCCGCGGTAA**  TTGTCAGGTGT**GTGYCAGCMGCCGCGGTAA** | This study |  |
| Fungi | 1st | ITS7F  ITS4R | ITS2 | ~310 | GTGARTCATCGAATCTTTG  TCCTCCGCTTATTGATATGC | Ihrmark et al., (2012)  White et al., (1990) |  |
|  | 2nd | Indexed  (n=85) | FI0001-ITS4r  FI0002-ITS4r  FI0003-ITS4r  FI0004-ITS4r  FI0005-ITS4r |  | TTAACCTTGGA**TCCTCCGCTTATTGATATGC**  TTAACCGAAGA**TCCTCCGCTTATTGATATGC**  TTAACGACAGA**TCCTCCGCTTATTGATATGC**  TTACTTACGGA**TCCTCCGCTTATTGATATGC**  TTACTTGTCGA**TCCTCCGCTTATTGATATGC** | This study |  |

**Supplementary Table S3.** PCR reagents and thermocycling conditions used for amplicon sequencing analysis.

| **PCR reaction** | | | |
| --- | --- | --- | --- |
| **Reagents** | **Volume (μl)** | **Concentration** | **Comments** |
| Primer F | 1 | 0.5 μΜ |  |
| Primer R | 1 | 0.5 μΜ |  |
| BSA | 0.4 | 0.4 μg/μl | Added only in the first amplification step |
| Polymerase Q5 (2x MasterMix) | 10 | 1x |  |
| ddH_2_O | 5.6 |  |  |
| DNA | 2 | 0.2 ng/μl |  |
| Total | 20 |  |  |
| **PCR conditions** | | | |
| **Step** | **Temperature (°C)** | **Time** | **Number of Cycles** |
| Initial Denaturation | 98 | 30 sec |  |
| Denaturation | 98 | 10 sec | 28 in the first amplification step /  7 in the second amplification step |
| Annealing | 50 for bacteria/ 55 for fungi | 30 sec |  |
| Extension | 72 | 30 sec |  |
| Final extension | 72 | 10 min |  |

**Supplementary Table S4.** PERMANOVA analysis of the fungal and bacterial wood microbiome (Signif. codes: 0.001 ‘***’ 0.01 ‘**’ 0.05 ‘*’)

| Factor | Degrees of Freedom | Sum of squares | F. Model | R^2^ (%) | P-value (>F) |  |
| --- | --- | --- | --- | --- | --- | --- |
| Fungi – All varieties | | | | | | |
| GTDs condition | 1 | 1.221 | 3.5576 | 3.5 | 0.001*** |  |
| Variety/Geographic Location | 8 | 7.902 | 7.2183 | 22.7 | 0.001*** |  |
| Residuals | 75 | 25.75 | 0.73838 |  |  |  |
| Fungi – Agiorgitiko | | | | | | |
| GTDs condition | 1 | 0.4414 | 1.8848 | 8.4 | 0.012* |  |
| Vineyard | 2 | 1.0777 | 2.3008 | 20.5 | 0.001*** |  |
| Residuals | 16 | 3.7473 | 0.71154 |  |  |  |
| Fungi – Xinomavro | | | | | | |
| GTDs condition | 1 | 1.5707 | 4.6644 | 13.9 | 0.001*** |  |
| Vineyard | 2 | 0.9678 | 1.437 | 8.6 | 0.038* |  |
| Residuals | 26 | 8.7552 | 0.77523 |  |  |  |
| Fungi – Vidiano | | | | | | |
| GTDs condition | 1 | 0.6186 | 1.6121 | 4.2 | 0.054 |  |
| Vineyard | 2 | 2.2099 | 2.8793 | 15 | 0.001*** |  |
| Residuals | 31 | 11.8962 | 0.80791 |  |  |  |
| Bacteria – All varieties | | | | | | |
| GTDs condition | 1 | 1.863 | 5.5443 | 5.2 | 0.001*** |  |
| Variety/Geographic Location | 8 | 9.169 | 10.3873 | 25.5 | 0.001*** |  |
| Residuals | 74 | 24.863 | 0.69267 |  |  |  |
| Bacteria – Agiorgitiko | | | | | | |
| GTDs condition | 1 | 0.3776 | 1.0526 | 5.3 | 0.263 |  |
| Vineyard | 2 | 0.9631 | 1.3423 | 13.6 | 0.001*** |  |
| Residuals | 16 | 5.7399 | 0.81065 |  |  |  |
| Bacteria - Xinomavro | | | | | | |
| GTDs condition | 1 | 2.2788 | 6.7866 | 19.7 | 0.001*** |  |
| Vineyard | 2 | 0.8801 | 1.3105 | 7.6 | 0.079 . |  |
| Residuals | 25 | 8.3943 | 0.72658 |  |  |  |
| Bacteria - Vidiano | | | | | | |
| GTDs condition | 1 | 0.6553 | 2.2694 | 6 | 0.007** |  |
| Vineyard | 2 | 1.3166 | 2.2799 | 12.1 | 0.006** |  |
| Residuals | 31 | 8.951 | 0.81947 |  |  | |

**Supplementary Table S5.** ASVs detected in the wood microbiome of the vines studied that are considered as putative pathogens involved in GTDs. Each ASV was phylogenetically assigned to the closest verified taxonomic level (genus or species)

| ASV | Cultivar | State | % | Phylum | Class | Order | Family | Genus | Species |
| --- | --- | --- | --- | --- | --- | --- | --- | --- | --- |
| ASV005 | Agiorgitiko | Asymptomatic | 8.61 | Ascomycota | Eurotiomycetes | Phaeomoniellales | Phaeomoniellaceae | Phaeomoniella | chlamydospora |
| ASV005 | Agiorgitiko | Symptomatic | 7.78 | Ascomycota | Eurotiomycetes | Phaeomoniellales | Phaeomoniellaceae | Phaeomoniella | chlamydospora |
| ASV005 | Xinomavro | Symptomatic | 1.80 | Ascomycota | Eurotiomycetes | Phaeomoniellales | Phaeomoniellaceae | Phaeomoniella | chlamydospora |
| ASV005 | Vidiano | Asymptomatic | 12.90 | Ascomycota | Eurotiomycetes | Phaeomoniellales | Phaeomoniellaceae | Phaeomoniella | chlamydospora |
| ASV005 | Vidiano | Symptomatic | 6.85 | Ascomycota | Eurotiomycetes | Phaeomoniellales | Phaeomoniellaceae | Phaeomoniella | chlamydospora |
| ASV008 | Agiorgitiko | Asymptomatic | 4.57 | Ascomycota | Sordariomycetes | Togniniales | Togniniaceae | Phaeoacremonium | iranianum |
| ASV008 | Agiorgitiko | Symptomatic | 7.46 | Ascomycota | Sordariomycetes | Togniniales | Togniniaceae | Phaeoacremonium | iranianum |
| ASV008 | Xinomavro | Asymptomatic | 0.03 | Ascomycota | Sordariomycetes | Togniniales | Togniniaceae | Phaeoacremonium | iranianum |
| ASV008 | Xinomavro | Symptomatic | 3.91 | Ascomycota | Sordariomycetes | Togniniales | Togniniaceae | Phaeoacremonium | iranianum |
| ASV008 | Vidiano | Asymptomatic | 5.15 | Ascomycota | Sordariomycetes | Togniniales | Togniniaceae | Phaeoacremonium | iranianum |
| ASV008 | Vidiano | Symptomatic | 3.43 | Ascomycota | Sordariomycetes | Togniniales | Togniniaceae | Phaeoacremonium | iranianum |
| ASV009 | Agiorgitiko | Asymptomatic | 1.10 | Ascomycota | Dothideomycetes | Botryosphaeriales | Botryosphaeriaceae | Diplodia | spp. |
| ASV009 | Agiorgitiko | Symptomatic | 3.87 | Ascomycota | Dothideomycetes | Botryosphaeriales | Botryosphaeriaceae | Diplodia | spp. |
| ASV009 | Xinomavro | Asymptomatic | 11.99 | Ascomycota | Dothideomycetes | Botryosphaeriales | Botryosphaeriaceae | Diplodia | spp. |
| ASV009 | Xinomavro | Symptomatic | 6.88 | Ascomycota | Dothideomycetes | Botryosphaeriales | Botryosphaeriaceae | Diplodia | spp. |
| ASV009 | Vidiano | Asymptomatic | 1.73 | Ascomycota | Dothideomycetes | Botryosphaeriales | Botryosphaeriaceae | Diplodia | spp. |
| ASV009 | Vidiano | Symptomatic | 1.26 | Ascomycota | Dothideomycetes | Botryosphaeriales | Botryosphaeriaceae | Diplodia | spp. |
| ASV011 | Agiorgitiko | Symptomatic | 6.02 | Basidiomycota | Agaricomycetes | Hymenochaetales | Hymenochaetaceae | Fomitiporia | spp. |
| ASV011 | Xinomavro | Asymptomatic | 0.02 | Basidiomycota | Agaricomycetes | Hymenochaetales | Hymenochaetaceae | Fomitiporia | spp. |
| ASV011 | Xinomavro | Symptomatic | 2.09 | Basidiomycota | Agaricomycetes | Hymenochaetales | Hymenochaetaceae | Fomitiporia | spp. |
| ASV011 | Vidiano | Asymptomatic | 0.51 | Basidiomycota | Agaricomycetes | Hymenochaetales | Hymenochaetaceae | Fomitiporia | spp. |
| ASV011 | Vidiano | Symptomatic | 5.26 | Basidiomycota | Agaricomycetes | Hymenochaetales | Hymenochaetaceae | Fomitiporia | spp. |
| ASV013 | Agiorgitiko | Asymptomatic | 7.64 | Ascomycota | Sordariomycetes | Xylariales | Amphisphaeriaceae | Seimatosporium | vitis |
| ASV013 | Agiorgitiko | Symptomatic | 1.99 | Ascomycota | Sordariomycetes | Xylariales | Amphisphaeriaceae | Seimatosporium | vitis |
| ASV013 | Xinomavro | Asymptomatic | 1.59 | Ascomycota | Sordariomycetes | Xylariales | Amphisphaeriaceae | Seimatosporium | vitis |
| ASV013 | Xinomavro | Symptomatic | 1.19 | Ascomycota | Sordariomycetes | Xylariales | Amphisphaeriaceae | Seimatosporium | vitis |
| ASV013 | Vidiano | Asymptomatic | 6.64 | Ascomycota | Sordariomycetes | Xylariales | Amphisphaeriaceae | Seimatosporium | vitis |
| ASV013 | Vidiano | Symptomatic | 2.07 | Ascomycota | Sordariomycetes | Xylariales | Amphisphaeriaceae | Seimatosporium | vitis |
| ASV014 | Agiorgitiko | Symptomatic | 2.98 | Ascomycota | Eurotiomycetes | Phaeomoniellales | Phaeomoniellaceae | Phaeomoniella | chlamydospora |
| ASV014 | Xinomavro | Symptomatic | 0.65 | Ascomycota | Eurotiomycetes | Phaeomoniellales | Phaeomoniellaceae | Phaeomoniella | chlamydospora |
| ASV014 | Vidiano | Asymptomatic | 4.03 | Ascomycota | Eurotiomycetes | Phaeomoniellales | Phaeomoniellaceae | Phaeomoniella | chlamydospora |
| ASV014 | Vidiano | Symptomatic | 6.23 | Ascomycota | Eurotiomycetes | Phaeomoniellales | Phaeomoniellaceae | Phaeomoniella | chlamydospora |
| ASV015 | Xinomavro | Symptomatic | 7.47 | Ascomycota | Dothideomycetes | Botryosphaeriales | Botryosphaeriaceae | Lasiodiplodia | spp. |
| ASV020 | Agiorgitiko | Symptomatic | 0.55 | Basidiomycota | Agaricomycetes | Hymenochaetales | Hymenochaetaceae | Fomitiporia | spp. |
| ASV020 | Xinomavro | Symptomatic | 3.38 | Basidiomycota | Agaricomycetes | Hymenochaetales | Hymenochaetaceae | Fomitiporia | spp. |
| ASV020 | Vidiano | Asymptomatic | 0.68 | Basidiomycota | Agaricomycetes | Hymenochaetales | Hymenochaetaceae | Fomitiporia | spp. |
| ASV021 | Agiorgitiko | Symptomatic | 3.82 | Basidiomycota | Agaricomycetes | Hymenochaetales | Hymenochaetaceae | Fomitiporia | spp. |
| ASV024 | Xinomavro | Asymptomatic | 1.61 | Ascomycota | Dothideomycetes | Botryosphaeriales | Botryosphaeriaceae | Botryosphaeria | spp. |
| ASV024 | Xinomavro | Symptomatic | 1.33 | Ascomycota | Dothideomycetes | Botryosphaeriales | Botryosphaeriaceae | Botryosphaeria | spp. |
| ASV024 | Vidiano | Asymptomatic | 0.51 | Ascomycota | Dothideomycetes | Botryosphaeriales | Botryosphaeriaceae | Botryosphaeria | spp. |
| ASV024 | Vidiano | Symptomatic | 10.41 | Ascomycota | Dothideomycetes | Botryosphaeriales | Botryosphaeriaceae | Botryosphaeria | spp. |
| ASV026 | Agiorgitiko | Asymptomatic | 0.16 | Ascomycota | Dothideomycetes | Botryosphaeriales | Botryosphaeriaceae | Neofusicoccum | spp. |
| ASV026 | Agiorgitiko | Symptomatic | 0.25 | Ascomycota | Dothideomycetes | Botryosphaeriales | Botryosphaeriaceae | Neofusicoccum | spp. |
| ASV026 | Vidiano | Asymptomatic | 1.56 | Ascomycota | Dothideomycetes | Botryosphaeriales | Botryosphaeriaceae | Neofusicoccum | spp. |
| ASV026 | Vidiano | Symptomatic | 5.84 | Ascomycota | Dothideomycetes | Botryosphaeriales | Botryosphaeriaceae | Neofusicoccum | spp. |
| ASV027 | Xinomavro | Symptomatic | 3.18 | Ascomycota | Sordariomycetes | Hypocreales | Hypocreaceae | Acremonium | alternatum |
| ASV027 | Vidiano | Symptomatic | 1.81 | Ascomycota | Sordariomycetes | Hypocreales | Hypocreaceae | Acremonium | alternatum |
| ASV029 | Agiorgitiko | Symptomatic | 2.13 | Basidiomycota | Agaricomycetes | Hymenochaetales | Hymenochaetaceae | Fomitiporia | spp. |
| ASV029 | Vidiano | Asymptomatic | 0.53 | Basidiomycota | Agaricomycetes | Hymenochaetales | Hymenochaetaceae | Fomitiporia | spp. |
| ASV032 | Xinomavro | Asymptomatic | 6.98 | Ascomycota | Dothideomycetes | Botryosphaeriales | Botryosphaeriaceae |  |  |
| ASV040 | Agiorgitiko | Symptomatic | 1.72 | Basidiomycota | Agaricomycetes | Hymenochaetales | Hymenochaetaceae | Fomitiporia | spp. |
| ASV041 | Agiorgitiko | Symptomatic | 2.08 | Basidiomycota | Agaricomycetes | Hymenochaetales | Hymenochaetaceae | Phellinus | rhamni |
| ASV042 | Xinomavro | Symptomatic | 1.52 | Basidiomycota | Agaricomycetes | Hymenochaetales | Hymenochaetaceae | Fomitiporia | spp. |
| ASV045 | Vidiano | Asymptomatic | 3.74 | Ascomycota | Sordariomycetes | Togniniales | Togniniaceae | Phaeoacremonium | sicilianum |
| ASV045 | Vidiano | Symptomatic | 0.84 | Ascomycota | Sordariomycetes | Togniniales | Togniniaceae | Phaeoacremonium | sicilianum |
| ASV046 | Agiorgitiko | Asymptomatic | 0.16 | Ascomycota | Dothideomycetes | Pleosporales | Phaeosphaeriaceae | Neosetophoma | rosarum |
| ASV046 | Agiorgitiko | Symptomatic | 0.45 | Ascomycota | Dothideomycetes | Pleosporales | Phaeosphaeriaceae | Neosetophoma | rosarum |
| ASV046 | Vidiano | Asymptomatic | 1.34 | Ascomycota | Dothideomycetes | Pleosporales | Phaeosphaeriaceae | Neosetophoma | rosarum |
| ASV046 | Vidiano | Symptomatic | 1.46 | Ascomycota | Dothideomycetes | Pleosporales | Phaeosphaeriaceae | Neosetophoma | rosarum |
| ASV052 | Agiorgitiko | Symptomatic | 1.63 | Basidiomycota | Agaricomycetes | Hymenochaetales | Hymenochaetaceae | Fomitiporia | spp. |
| ASV053 | Agiorgitiko | Symptomatic | 1.02 | Basidiomycota | Agaricomycetes | Hymenochaetales | Hymenochaetaceae | Fomitiporia | spp. |
| ASV058 | Agiorgitiko | Asymptomatic | 0.12 | Ascomycota | Dothideomycetes | Pleosporales | Phaeosphaeriaceae | Neosetophoma | spp. |
| ASV058 | Vidiano | Asymptomatic | 2.06 | Ascomycota | Dothideomycetes | Pleosporales | Phaeosphaeriaceae | Neosetophoma | spp. |
| ASV058 | Vidiano | Symptomatic | 1.03 | Ascomycota | Dothideomycetes | Pleosporales | Phaeosphaeriaceae | Neosetophoma | spp. |
| ASV063 | Agiorgitiko | Symptomatic | 0.55 | Basidiomycota | Agaricomycetes | Hymenochaetales | Hymenochaetaceae | Fomitiporia | spp. |
| ASV063 | Xinomavro | Symptomatic | 1.12 | Basidiomycota | Agaricomycetes | Hymenochaetales | Hymenochaetaceae | Fomitiporia | spp. |
| ASV065 | Xinomavro | Symptomatic | 0.26 | Ascomycota | Leotiomycetes | Helotiales | Dermateaceae | Neofabraea | spp. |
| ASV065 | Vidiano | Symptomatic | 1.11 | Ascomycota | Leotiomycetes | Helotiales | Dermateaceae | Neofabraea | spp. |
| ASV068 | Agiorgitiko | Asymptomatic | 0.15 | Ascomycota | Dothideomycetes | Pleosporales | Didymosphaeriaceae | Kalmusia | variispora |
| ASV068 | Agiorgitiko | Symptomatic | 0.19 | Ascomycota | Dothideomycetes | Pleosporales | Didymosphaeriaceae | Kalmusia | variispora |
| ASV068 | Xinomavro | Symptomatic | 0.18 | Ascomycota | Dothideomycetes | Pleosporales | Didymosphaeriaceae | Kalmusia | variispora |
| ASV068 | Vidiano | Symptomatic | 1.07 | Ascomycota | Dothideomycetes | Pleosporales | Didymosphaeriaceae | Kalmusia | variispora |
| ASV072 | Xinomavro | Symptomatic | 1.61 | Ascomycota | Sordariomycetes | Togniniales | Togniniaceae | Phaeoacremonium | spp. |
| ASV073 | Agiorgitiko | Symptomatic | 0.48 | Basidiomycota | Agaricomycetes | Hymenochaetales | Hymenochaetaceae | Fomitiporia | spp. |
| ASV073 | Vidiano | Symptomatic | 1.22 | Basidiomycota | Agaricomycetes | Hymenochaetales | Hymenochaetaceae | Fomitiporia | spp. |
| ASV074 | Vidiano | Symptomatic | 2.13 | Ascomycota | Sordariomycetes | Diaporthales | Diaporthaceae | Diaporthe | spp. |
| ASV075 | Xinomavro | Asymptomatic | 0.08 | Ascomycota | Dothideomycetes | Pleosporales | Phaeosphaeriaceae | Neosetophoma | spp. |
| ASV075 | Xinomavro | Symptomatic | 1.39 | Ascomycota | Dothideomycetes | Pleosporales | Phaeosphaeriaceae | Neosetophoma | spp. |
| ASV075 | Vidiano | Symptomatic | 0.14 | Ascomycota | Dothideomycetes | Pleosporales | Phaeosphaeriaceae | Neosetophoma | spp. |
| ASV076 | Xinomavro | Symptomatic | 0.30 | Ascomycota | Sordariomycetes | Hypocreales | Hypocreaceae | Acremonium | sordidulum |
| ASV076 | Vidiano | Symptomatic | 0.79 | Ascomycota | Sordariomycetes | Hypocreales | Hypocreaceae | Acremonium | sordidulum |
| ASV091 | Agiorgitiko | Asymptomatic | 0.70 | Ascomycota | Eurotiomycetes | Phaeomoniellales | Phaeomoniellaceae | Phaeomoniella | spp. |
| ASV091 | Agiorgitiko | Symptomatic | 0.25 | Ascomycota | Eurotiomycetes | Phaeomoniellales | Phaeomoniellaceae | Phaeomoniella | spp. |
| ASV093 | Vidiano | Symptomatic | 2.09 | Basidiomycota | Agaricomycetes | Hymenochaetales | Hymenochaetaceae | Fomitiporia | spp. |
| ASV098 | Vidiano | Asymptomatic | 1.22 | Ascomycota | Sordariomycetes | Togniniales | Togniniaceae | Phaeoacremonium | spp. |
| ASV103 | Agiorgitiko | Symptomatic | 0.45 | Basidiomycota | Agaricomycetes | Hymenochaetales | Hymenochaetaceae | Phellinus | rhamni |
| ASV104 | Xinomavro | Asymptomatic | 0.19 | Ascomycota | Dothideomycetes | Botryosphaeriales | Botryosphaeriaceae | Lasiodiplodia | spp. |
| ASV104 | Xinomavro | Symptomatic | 0.78 | Ascomycota | Dothideomycetes | Botryosphaeriales | Botryosphaeriaceae | Lasiodiplodia | spp. |
| ASV118 | Vidiano | Asymptomatic | 1.14 | Ascomycota | Sordariomycetes | Togniniales | Togniniaceae | Phaeoacremonium | iranianum |
| ASV131 | Xinomavro | Symptomatic | 0.71 | Basidiomycota | Agaricomycetes | Hymenochaetales | Hymenochaetaceae | Fomitiporia | spp. |
| ASV134 | Xinomavro | Symptomatic | 1.33 | Basidiomycota | Agaricomycetes | Hymenochaetales | Hymenochaetaceae | Fomitiporia | spp. |
| ASV159 | Xinomavro | Asymptomatic | 0.03 | Ascomycota | Dothideomycetes | Pleosporales | Phaeosphaeriaceae | Neosetophoma | spp. |
| ASV159 | Xinomavro | Symptomatic | 0.47 | Ascomycota | Dothideomycetes | Pleosporales | Phaeosphaeriaceae | Neosetophoma | spp. |
| ASV163 | Xinomavro | Symptomatic | 0.37 | Ascomycota | Dothideomycetes | Pleosporales | Phaeosphaeriaceae | Neosetophoma | spp. |
| ASV168 | Xinomavro | Symptomatic | 0.51 | Basidiomycota | Agaricomycetes | Hymenochaetales | Hymenochaetaceae | Fomitiporia | spp. |
| ASV179 | Agiorgitiko | Asymptomatic | 0.41 | Ascomycota | Eurotiomycetes | Phaeomoniellales | Phaeomoniellaceae | Phaeomoniella | spp. |
| ASV202 | Vidiano | Asymptomatic | 0.65 | Basidiomycota | Agaricomycetes | Hymenochaetales | Hymenochaetaceae | Fomitiporia | spp. |
| ASV210 | Agiorgitiko | Asymptomatic | 0.30 | Ascomycota | Eurotiomycetes | Phaeomoniellales | Phaeomoniellaceae | Phaeomoniella | spp. |
| ASV216 | Agiorgitiko | Symptomatic | 0.18 | Ascomycota | Dothideomycetes | Botryosphaeriales | Botryosphaeriaceae | Dothiorella | rosulata |
| ASV223 | Vidiano | Asymptomatic | 0.27 | Ascomycota | Sordariomycetes | Togniniales | Togniniaceae | Phaeoacremonium | iranianum |
| ASV232 | Xinomavro | Symptomatic | 0.29 | Ascomycota | Dothideomycetes | Pleosporales | Phaeosphaeriaceae | Neosetophoma | spp. |
| ASV270 | Vidiano | Symptomatic | 0.20 | Ascomycota | Dothideomycetes | Botryosphaeriales | Botryosphaeriaceae | Diplodia | spp. |
| ASV278 | Agiorgitiko | Asymptomatic | 0.10 | Ascomycota | Sordariomycetes | Xylariales | Diatrypaceae | Eutypa | spp. |
| ASV278 | Vidiano | Symptomatic | 0.12 | Ascomycota | Sordariomycetes | Xylariales | Diatrypaceae | Eutypa | spp. |
| ASV282 | Vidiano | Symptomatic | 0.24 | Ascomycota | Dothideomycetes | Botryosphaeriales | Botryosphaeriaceae | Neofusicoccum | spp. |
| ASV319 | Xinomavro | Asymptomatic | 0.03 | Ascomycota | Dothideomycetes | Botryosphaeriales | Botryosphaeriaceae |  |  |
| ASV325 | Vidiano | Symptomatic | 0.19 | Ascomycota | Sordariomycetes | Hypocreales | Hypocreales_fam_Incertae | Acremonium | fusidioides |
| ASV338 | Agiorgitiko | Asymptomatic | 0.21 | Ascomycota | Sordariomycetes | Xylariales | Amphisphaeriaceae | Seimatosporium | vitis |
| ASV339 | Vidiano | Asymptomatic | 0.63 | Ascomycota | Sordariomycetes | Xylariales | Amphisphaeriaceae | Seimatosporium | vitis |
| ASV341 | Xinomavro | Symptomatic | 0.18 | Ascomycota | Sordariomycetes | Xylariales | Amphisphaeriaceae | Seimatosporium | vitis |
| ASV552 | Xinomavro | Symptomatic | 0.22 | Ascomycota | Sordariomycetes | Xylariales | Diatrypaceae | Eutypa | crustata |
| ASV659 | Vidiano | Asymptomatic | 0.15 | Ascomycota | Dothideomycetes | Pleosporales | Didymosphaeriaceae | Didymosphaeria | futilis |
| ASV677 | Vidiano | Symptomatic | 0.37 | Ascomycota | Dothideomycetes | Pleosporales | Phaeosphaeriaceae | Neosetophoma | spp. |
| ASV803 | Vidiano | Asymptomatic | 0.12 | Ascomycota | Eurotiomycetes | Phaeomoniellales |  |  |  |
| ASV805 | Vidiano | Asymptomatic | 0.12 | Ascomycota | Sordariomycetes | Xylariales | Diatrypaceae | Eutypa | tetragona |
| ASV806 | Vidiano | Asymptomatic | 0.09 | Ascomycota | Dothideomycetes | Botryosphaeriales |  |  |  |
